# Fact boxes that inform individual decisions may contribute to a more positive evaluation of COVID-19 vaccinations at the population level

**DOI:** 10.1371/journal.pone.0274186

**Published:** 2022-09-12

**Authors:** Felix G. Rebitschek, Christin Ellermann, Mirjam A. Jenny, Nico A. Siegel, Christian Spinner, Gert G. Wagner

**Affiliations:** 1 Harding Center for Risk Literacy, Faculty of Health Sciences Brandenburg, University of Potsdam, Potsdam, Brandenburg, Germany; 2 Max Planck Institute for Human Development, Berlin, Germany; 3 Health Communication Research Group, Implementation Science, Bernhard Nocht Institute for Tropical Medicine, Hamburg, Germany; 4 Media and Communication Science, University of Erfurt, Erfurt, Thüringen, Germany; 5 Infratest dimap, Berlin, Germany; 6 German Socio-Economic Panel Study (SOEP), Berlin, Germany; 7 Federal Institute for Population Research, Wiesbaden, Hessen, Germany; INSERM CEA Cognitive Neuroimaging Unit, FRANCE

## Abstract

**Objective:**

For an effective control of the SARS-CoV-2 pandemic with vaccines, most people in a population need to be vaccinated. It is thus important to know how to inform the public with reference to individual preferences–while also acknowledging the societal preference to encourage vaccinations. According to the health care standard of informed decision-making, a comparison of the benefits and harms of (not) having the vaccination would be required to inform undecided and skeptical people. To test evidence-based fact boxes, an established risk communication format, and to inform their development, we investigated their contribution to knowledge and evaluations of COVID-19 vaccines.

**Methods:**

We conducted four studies (1, 2, and 4 were population-wide surveys with N = 1,942 to N = 6,056): Study 1 assessed the relationship between vaccination knowledge and intentions in Germany over three months. Study 2 assessed respective information gaps and needs of the population in Germany. In parallel, an experiment (Study 3) with a mixed design (presentation formats; pre-post-comparison) assessed the effect of fact boxes on risk perceptions and fear, using a convenience sample (N = 719). Study 4 examined how effective two fact box formats are for informing vaccination intentions, with a mixed experimental design: between-subjects (presentation formats) and within-subjects (pre-post-comparison).

**Results:**

Study 1 showed that vaccination knowledge and vaccination intentions increased between November 2020 and February 2021. Study 2 revealed objective information requirements and subjective information needs. Study 3 showed that the fact box format is effective in adjusting risk perceptions concerning COVID-19. Based on those results, fact boxes were revised and implemented with the help of a national health authority in Germany. Study 4 showed that simple fact boxes increase vaccination knowledge and positive evaluations in skeptics and undecideds.

**Conclusion:**

Fact boxes can inform COVID-19 vaccination intentions of undecided and skeptical people without threatening societal vaccination goals of the population.

## Introduction

Effective vaccination strategies against the SARS-CoV-2 pandemic require a large proportion of the population to have the vaccine [1]. In December 2020, when the first vaccine was approved, about 37 to 45% of the adult population in Germany definitely intended to get vaccinated, according to population-wide national polls [2, 3]. In December 2021, vaccination rates in Germany approximated a plateau below 85% of people aged between 18 and 59 years, and below 90% of people 60 years and older, respectively [4]. The vaccinated population included a large share of those who were undecideds (who initially did not show a preference for or against vaccination) and skeptics (who more likely would not have had a vaccination initially but were not categorically opposed to it) before (in sum about 24 to 25% of the adult population between April and December 2020 [5, 6]). Before vaccines became available at the end of 2020, undecideds and skeptics sought information to make vaccination decisions [7, 8]. In fact, however, persuasive measures–including political, societal, moral, and social pressure–that do not serve to inform citizens played a substantial role in these decisions population-wide [9].

Health communication that informs and potentially convinces undecideds and skeptics about medical countermeasures during an emergency [10] represents an alternative to persuasive strategies that threaten trustworthiness and credibility of the communicator and of vaccinations [11]. According to international standards of evidence-based health care [12] and the patient protection law in Germany [13]–along with the ethical ideal of evidence-based decision-making [14]–every citizen should be enabled to weigh the possible benefits and harms of medical options on the basis of the best available evidence and to decide freely on this basis (informed decision-making) [15]. Accordingly, the information communicated should enable undecideds and skeptics to compare the benefits and harms of having or not having the vaccination.

The objective of our studies was to investigate how a public health information intervention (“mRNA COVID-19 vaccination fact boxes”) that was jointly developed by the Harding Center for Risk Literacy and the Robert Koch Institute (RKI) might shape the evaluation of COVID-19 vaccination by means of balanced information. Because the best available evidence clearly shows effectiveness (preventing COVID-19 and related hospitalization) and safety (with limited severe side effects), inferred benefit-harm ratios may be convincing. Thus, informed decision making, which considers individual preferences, more likely encourages vaccination and is expected to result in a net surplus of proponents over opponents at the population level. The expected surplus would be in line with the societal preference to increase the vaccinating share of the population in order to protect public health.

A “fact box” is a tabular or graphical version of a balance sheet [16] that summarizes the best available evidence on the benefits and harms of medical options and how likely these will occur [17, 18]. Fact boxes inform various health decisions, including those about medical treatments, cancer screenings, and vaccinations [17, 19, 20]. In contrast to regulation, incentives, and (invisible) nudges [21], fact boxes are not designed to enforce directed behavioral change [22]. They are boosts that have been shown to enable comprehension of medical risks and options and help acquire short-term knowledge thereof [23, 24].

We hypothesized that an intervention with a COVID-19 vaccination fact box not only conveys knowledge and corrects risk perceptions but also can support positive COVID-19 vaccination evaluations without employing persuasion. We conducted four studies to inform the further development of a COVID-19 vaccine fact box and to evaluate its impact on vaccination knowledge and evaluation. First, we assessed the relationship between vaccination knowledge and intention in Germany (Study 1) to determine whether such knowledge potentially contributes to the intention to vaccinate. To inform further development of a COVID-19 vaccination fact box, we identified information requirements and needs of the population in Germany (Study 2). Because accurate risk perception is a prerequisite for informed decision-making [25], Study 3 –conducted in parallel to study 2 –tested whether the fact box improves COVID-19 risk perception, as compared with conventional information. Study 4 evaluated whether different fact box formats are effective for enabling informed vaccination intentions. Studies 1, 2, and 4 were population-wide surveys, each sampling from a multi-level stratified sample from a pool of 72,000 panelists who belonged to the 25 million members of the German PAYBACK customer loyalty program (S1 Fig).

## Study 1

To provide a foundation for our plan to promote COVID-19 vaccination knowledge, we assessed the relationship between vaccination knowledge and acceptance in Germany over a three-month period that included introduction of the first vaccines. According to the 5C model [26], which describes the five psychological antecedents of vaccination (confidence in vaccines, structural and psychological constraints, complacency in disease risk perception, collective responsibility in protecting others), the motivation to search for vaccination information is often linked to negative vaccination intention (calculation). On the other hand, vaccination knowledge can be linked to higher vaccination intentions [27] and can close the gap between intentions and actual vaccination decisions [28].

### Materials and methods

Survey data from the company Infratest dimap served as the basis for a secondary analysis of vaccination intentions between November 2020 and February 2021. As part of the company’s representative Coronavirus Online Panel Survey Special (Corona-Online-Meinungs-Panel-Survey Spezial, COMPASS), German citizens’ attitudes toward COVID-19 policy, perceptions of constraints, and general attitudes during the pandemic were surveyed. The survey was supplemented by questions on vaccination knowledge. Data collection by the company was exempt from institutional review board approval according to the guidelines of the German Psychological Society DGPS (Deutsche Gesellschaft für Psychologie) [29]. It was reasonable to assume that participation in the commercial survey did not produce harm or discomfort beyond everyday experience. Participants gave written consent to participate and also provided written consent when they registered for repeated multi-topic surveys (e.g. topics such as policy or health), receiving obligatory information on data protection. The study was conducted consistent with the Declaration of Helsinki. The study was not pre-registered.

#### Participants

The cross-sectional data were obtained from daily surveys of members of the German population [30]. For Study 1, independent samples with (T_1_, T_3_) and without (T_0_, T_2_, T_4_) knowledge assessment were conducted between 25 November 2020 and 16 February 2021. We do not provide a longitudinal sample analysis because there were different samples across assessments (S1 Fig). Between 13,664 and 13,816 German-speaking respondents with German citizenship were invited per assessment (about 300 per day). Between 14 and 46 of them were not eligible. Among those who were eligible, between 25.4% and 28.0% were non-responders in each case, between 1.0% and 1.5% did not complete the survey, and N = 2,037 (T_0_), N = 2,090 (T_1_), N = 4,021 (T_2_), N = 6,056 (T_3_), and N = 1,942 (T_4_) were presented with our items. Of the participants, 50.4% to 50.5% were female. They had an average age of 47.2 to 52.1 years, between 31.9% and 36.8% had a university entrance qualification, and about the half of them lived in places with less than 50,000 inhabitants and earned less than 3,500 euros per household per month (S1 Table). Our samples are representative of German citizens who are active online (for details see [31]): The samples were drawn by simple random sampling. To take the varying commitment of different groups in study participation into account (non-response bias), the samples were weighted according to demographic population features. Only data of participants who completed the survey were analyzed. Participants received remuneration worth about 1 euro.

#### Procedure

After their informed consent to multi-topic study participation, participants received demographic questions, questions about pandemic conditions, an inquiry on their experiences with COVID-19, and a request to evaluate non-pharmaceutical interventions, (e.g. mask wearing obligations) followed by questions on vaccination intention, evaluation, and knowledge items.

#### Measures

The outcomes of interest in Study 1 were vaccination intention and vaccination knowledge.

Vaccination intention was assessed with a single-option choice (“Vaccines against the coronavirus (COVID-19) are now available. If you get the chance, will you get vaccinated against COVID-19?”: Definitely yes, probably yes, probably not, definitely not, I cannot yet say / am still undecided, I am already vaccinated; the last option was introduced when vaccines were available, from T_2_ onwards).

Vaccination knowledge was assessed with a focus on vaccination decisions: four items on potential harms (e.g. headache with and without vaccine), and uncertainty (e.g. reduction of contagiousness). The responses were scored according to the best available evidence in December 2020 (S3 Table). Respondents’ estimates of how many people get sick with COVID-19 if vaccinated or not after meeting an infected person were elicited using a normalized frequency format (out of 1,000 people) to show understanding of vaccine efficacy. Resulting inferences reflected an underlying risk ratio between the estimations. To avoid zero in the denominator, the division numerator and denominator were adjusted with +1 out of 1,000 (88% to 98% vaccine efficacy was scored as correct based on the results of a meta-analysis, published on 4 February 2021) [32]. For correct answers, we tolerated a +-2% margin of error.

#### Analysis

Descriptive analyses, linear and logistic regressions, and Spearman rank correlation analyses of vaccination intentions and knowledge scores were conducted.

### Results

**Fig 1:**
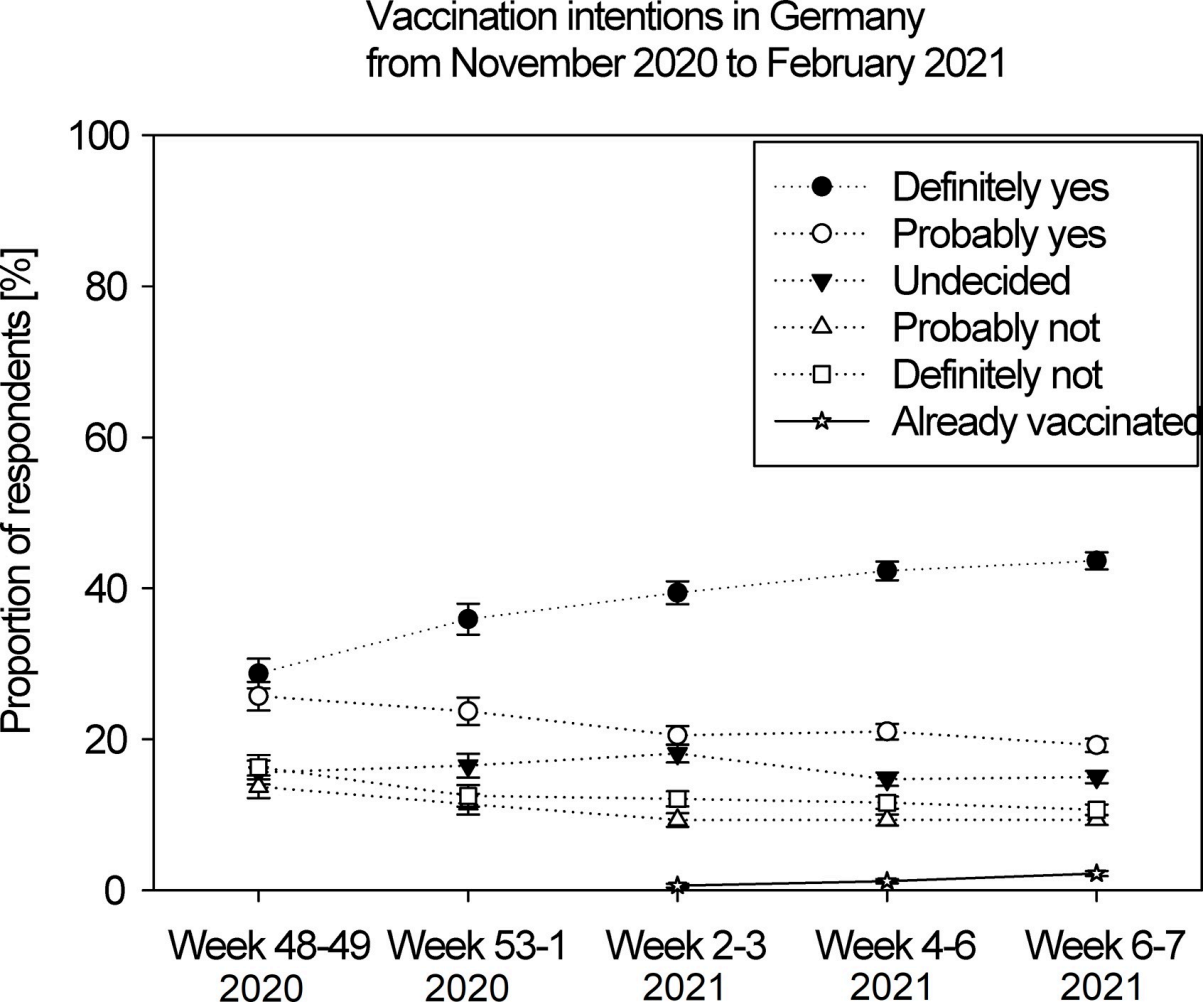
Proportion of respondents according to their intention to get vaccinated against COVID-19 or indicating that they already had. Independent samples were weighted at the time of their assessment. Error bars show 95% confidence intervals.

The cross-sectional analysis of five assessments showed increasing vaccination intentions between November 2020 and February 2021 in Germany. The cumulative proportion of proponents (probably or definitely intending to get the vaccination) grew from 54.4% (T_0_, end of Nov) to 65.1% (T_4_, mid-February, including 2.2% already vaccinated) (Fig 1). Each assessment after T_0_ made a proponent on average 1.1 times (odds ratio [OR]) more likely (95%-confidence interval [CI] [1.07, 1.12]), *χ*^2^(1) = 48.55, *p* < .001, although the proportion of those who probably intended to get vaccinated decreased (*p* < .001). At the same time, the proportion of undecideds declined (*p* = .044). The proportion of opponents (“definitely not”) and skeptics (“probably not”) decreased until actual vaccinations began (*p* < .001), but remained stable in January and February (*p* = .228), at about 12% and 9%, respectively.

**Fig 2:**
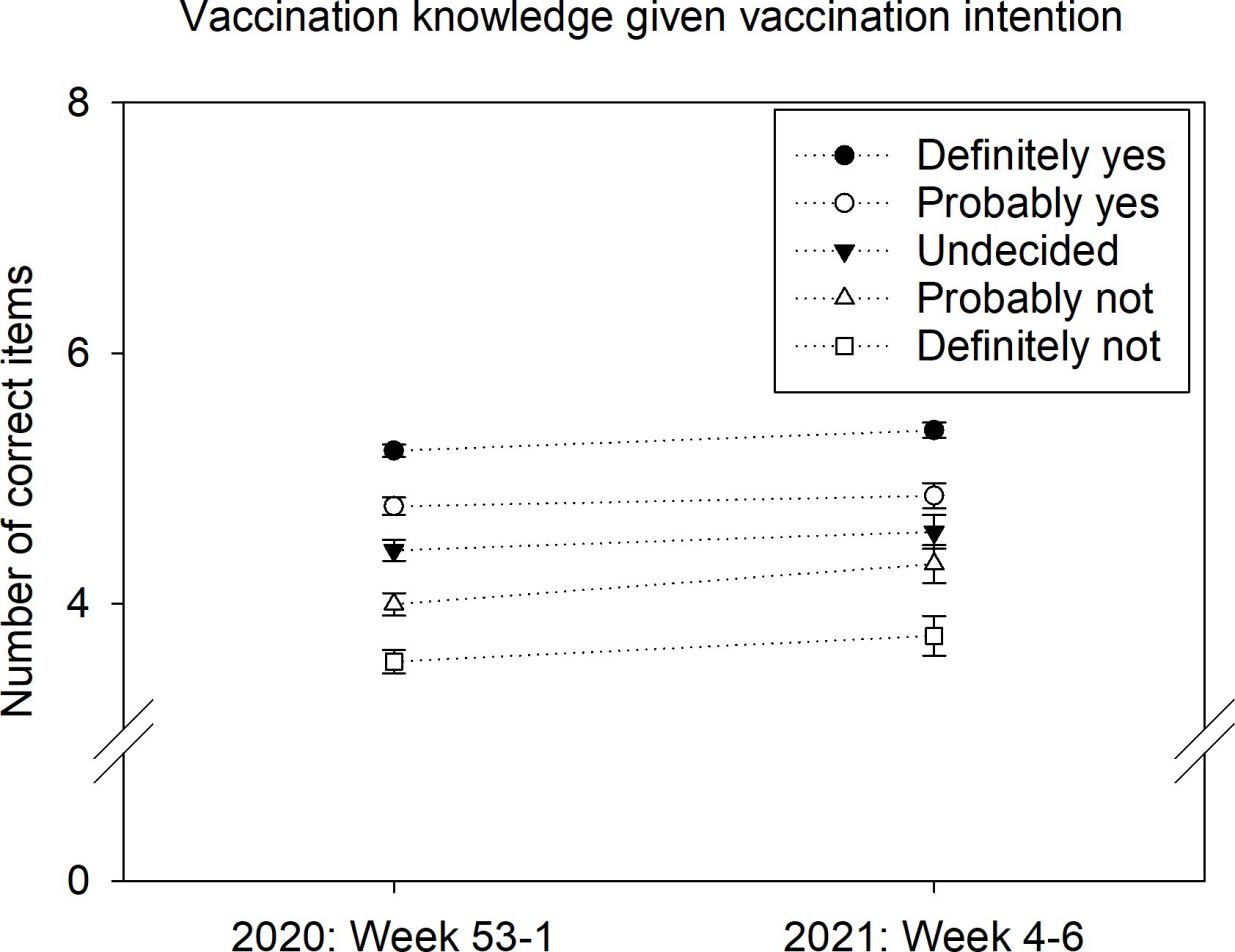
Number of correct items increased over two months according to respondents’ intention to get vaccinated against COVID-19. The independent samples were weighted at the time of their assessment. Error bars show the standard error of the mean.

Furthermore, between the end of December 2020 and the beginning of February 2021 (when only mRNA vaccines were available in Germany) the samples surveyed showed increasing vaccination knowledge, *F*(1,5034) = 40.81, *p* < .001, *β*_standardized_ = 0.09 (Fig 2). Over this period, those with low educational qualification (no formal qualification, primary, or secondary school) showed a similar increase in vaccination knowledge (from mean (M) = 4.40, standard error (SE) = 0.08 to M = 4.67, SE = 0.07; *p* < .001) compared with those with higher educational qualification (university entrance qualification) (from M = 4.68, SE = 0.04 to 4.96, SE = 0.03, *p* = .001).

More positive vaccination intentions were associated with higher knowledge scores (correlation coefficients *r*_T1_ = .36 and *r*_T3_ = .36, each *p* < .001). Among those who were undecided in February, little improvement in knowledge could be observed when compared with undecideds at the turn of the year (+0.1 correct out of 4 test items about vaccine-related uncertainty; compared with +0.3 among proponents).

### Discussion

From one month before first vaccine’s approval in Germany to two months afterwards, knowledge about the benefits and harms of vaccination increased, as did vaccination intentions. Although this finding cannot be extrapolated to the population’s knowledge development over the further course of the pandemic, it supports our assumption that improving vaccination knowledge could contribute to increasing vaccination rates. To improve vaccination knowledge, together with the RKI, the national health authority for disease surveillance and prevention, we developed evidence-based health information in the form of fact boxes.

## Study 2

The development process of evidence-based health information ideally involves patients and consumers [33]. First, it is essential to identify and to consider objective informational requirements, as identified by experts [34]: What information do recipients require? What do they not know? What false beliefs do they hold? Second, subjective information needs must be elicited and considered, not only with regard to decision-making but also with regard to developing trustworthy information, which may be unrelated to the expert’s view on which information is crucial. On the basis of a secondary analysis of data on perceived reasons for and against vaccination, we assessed the respective information requirements and needs of the population in Germany.

### Materials and methods

The study was conducted according to the conditions described under Study 1. The study was not pre-registered.

#### Participants

Study 2 was based on the sample T_0_ (N = 2,037) from the end of November 2020, described under Study 1 (S1 Table).

#### Design

We used the cross-sectional data from the survey described under Study 1.

#### Procedure

In the multi-topic survey, reasons were elicited after an inquiry on experiences with COVID-19 and a request to evaluate non-pharmaceutical interventions.

#### Measures

To identify required and needed information about the COVID-19 vaccination, outcomes of interest in Study 2 were reasons for and against having the vaccination.

Reasons in favor of or against COVID-19 vaccination were asked, with single-choice and open-response items depending on vaccination intention (e.g. for skeptics and opponents: “Why would you not want to get vaccinated if necessary?”).

#### Analysis

To analyze open responses about reasons in favor of and against COVID-19 vaccination, category systems starting with the subgroup of 18- to 39-year-olds were inductively developed independently by two researchers (one was the author C.E.). Successively, generated codes were reduced and summarized according to the 5C scale [26], a tool to monitor psychological antecedents of vaccination that describes five key elements: confidence (e.g. in the effectiveness and safety of vaccination, of the health care system), complacency (perception of risk), constraints (barriers to execution), calculation (extent of information seeking), and collective responsibility (sense of responsibility for the community). Afterward, the raters compared and consensually agreed on a combined category system with consistent codes for each item (S5 Table) and coded the responses of the three items again independently from each other. Interrater reliability was high, Cohen’s kappa = .92 (motivation), kappa = .90 (against vaccination), and kappa = .87 (undecided), respectively. Discrepancies in the coding of the individual answers were discussed, a uniform coding was jointly decided upon, and the codes were quantified.

### Results

Beyond the answers to closed questions about reasons against vaccination (36%: no threat of a severe COVID-19 course, 20%: pandemic passing without larger harm; S6 Table), we identified mitigators and facilitators for COVID-19 vaccination communication using open-ended questions: objective information requirements (experts agree that these appertain to everyone making an informed decision or in need of correction, e.g. false beliefs) and subjective information needs of the target population (these are personal and can be absolutely unrelated to experts’ views on informed decision-making, e.g. desired facts about who regulates the vaccine manufacturers).

Predominantly, undecided respondents’ motivations to get vaccinated were related to confidence in the vaccines and the delivering system, such as more medical research (36%), exclusion of harms (14%; more often from 40 years of age onward), and long-lasting high efficacy (7%). Besides those requirements, about 11% of undecideds below 40 years of age explicitly claimed their motivation to depend on more or better information. Motivations of those below 40 were more likely extrinsic (e.g., no contact restrictions, freedom to travel) but also reflected collective responsibility (18% stated that they would agree to vaccination if that would protect others, but only 7% of those aged 40–59 and 4% over 60 mentioned the same).

Nearly all reasons against vaccination named by undecideds, skeptics, and opponents showed information needs related to confidence and trust (S7 Table): belief in insufficient research on the vaccine and uncertainty about its efficacy and safety (28–52%, increasing with age), fear of harms (34–49%, decreasing with age), and distrust of policies or the vaccine (11–21%). Personal requirements (8%) and low disease risk perception (7%) played minor roles.

### Discussion

Our analysis of open-ended responses showed that confidence-related information needs concerning vaccine efficacy, safety, short- and long-term reactions and harms, and uncertainty are the most essential targets of vaccination communication. This is directly in line with information requirements of health information guidelines [35] and with the aim of building trust.

**Fig 3:**
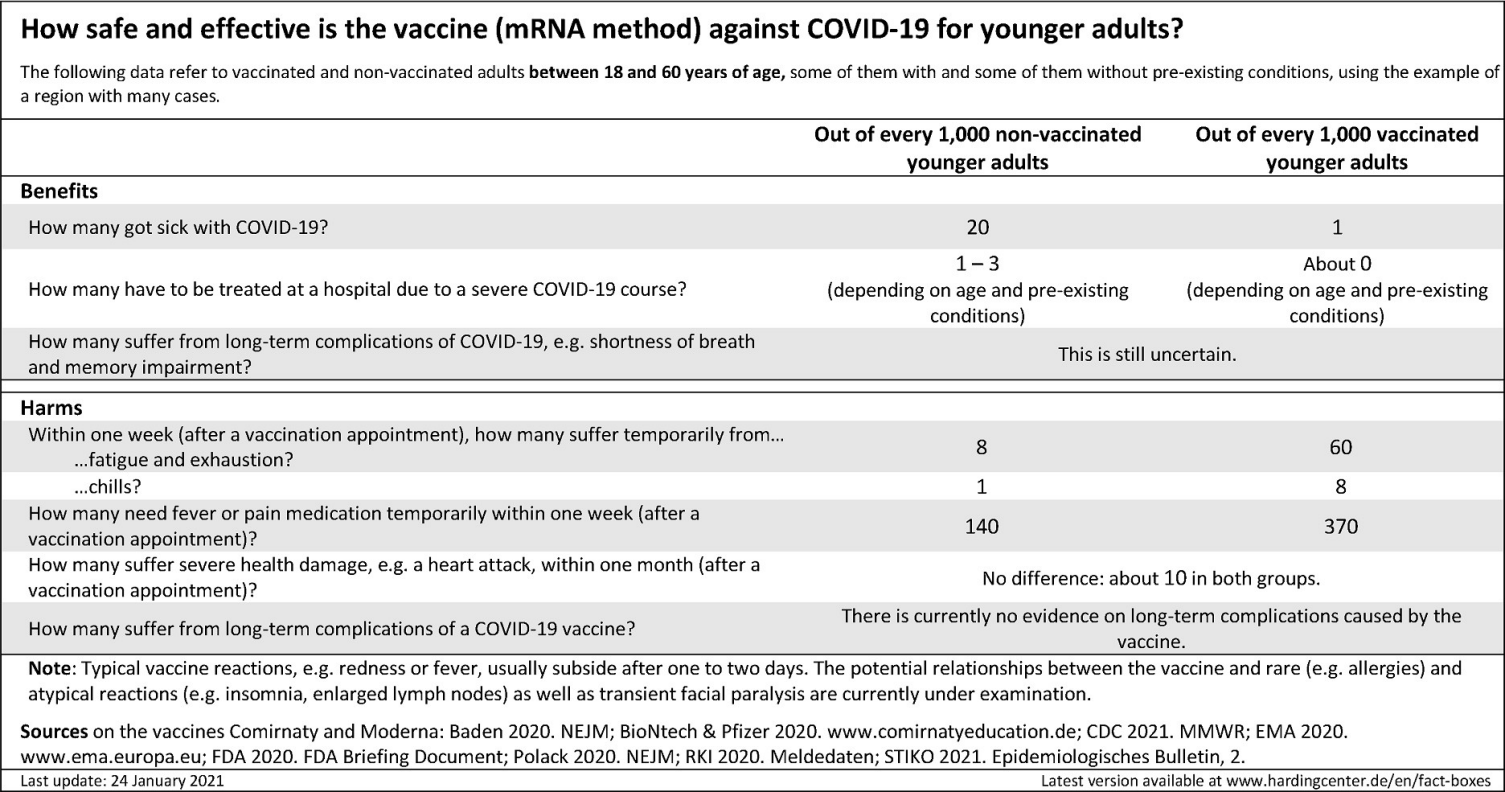
English translation of the simple fact box for people between the ages of 18 and 60 from Study 4.

In parallel to Study 2, a pilot format of the fact box was tested in Study 3 with regard to its effect on risk perception. Insights from both studies were used to inform the further development of a COVID-19 vaccination fact box (from S2A, S2B Fig to Fig 3 and S4–S6 Figs, e.g. by collapsing typical vaccination reactions).

## Study 3

Accurate risk perceptions of having or not having an intervention is a prerequisite of informed choices [25]. Although higher disease risk perceptions can be associated with higher vaccination intentions [36–38], overstating the risk of COVID-19 in vaccination communication can not only create fears but can also be detrimental to trustworthiness of medical and scientific experts, which in turn predicts uptake [36]. By communicating transparently and correcting false risk perceptions, fact boxes theoretically can inform the public without undermining trust in the communicating institution. Our online study with a convenience sample controlled whether fact boxes–as evidence-based health information that helps understand the risks of medical options [23, 24]–improve COVID-19 risk perception.

### Materials and methods

Ethical approval was obtained from the ethics committee of the University of Potsdam (Germany; 80/2020). The study was not pre-registered.

#### Participants

Seven hundred and nineteen adults aged 18 to 68 years (M = 28.8, SD = 8.8), who were recruited as a convenience sample through the online portal Prolific.co, completed Study 3 (demographics in S2 Table). Participants were eligible if their mother tongue was German and if Germany was their current country of residence. Participants were contacted by email with information about the study and a link to the online survey. They were remunerated with 2.65 British pounds (about 3 euros).

#### Design

Study 3 had a mixed experimental design: between-subjects variations (random assignment of each participant to one out of six presentation formats) and within-subjects analysis (pre- and post-presentation). The three presentation conditions were (1) vaccine fact box pilot (n = 120, “In case of contact with the coronavirus,” S2A Fig), (2) vaccine fact box pilot with social framing (n = 123, “If you spread the coronavirus,” S2B Fig), and (3) a control condition (n = 116; website from www.helios-gesundheit.de [39]), in which standard information on SARS-CoV-2, COVID-19 (without vaccination) and influenza from the internet was presented (between November 20 and February 21 among the top three German search findings comparing SARS-CoV-2 and influenza, S9 File). Three further presentation conditions for another experiment on disease risks are not reported here. Participants were not aware of the alternative formats. The same introductory text was provided for each condition.

#### Procedure

After giving informed consent and responding to demographic questions, participants answered items on disease risk perception and fear, on previous adherence to COVID-19 measures, and on vaccination intention and evaluation (baseline assessment). Then they were presented with the intervention (e.g. fact boxes). After reading the presentation formats with evaluation items (e.g. trust in the information presented), the intervention was removed and questions on fear, risk perception, adherence to non-pharmaceutical interventions (e.g. wearing masks, not reported here), and vaccination intentions and evaluations were repeated (post-assessment).

#### Measures

Outcomes of interest in Study 3 were risk perception, fear of getting or spreading COVID-19, and vaccination intention.

Risk perception and fear. Two items measured the fear of getting or spreading COVID-19 (“How greatly do you fear contracting COVID-19/infecting other adults?: Please use a scale from 0 to 10. A value of 0 means no fear at all and a value of 10 means a lot of fear. You can also use one of the values in between to indicate your rating.”). One item measured perceived severity (“How severe would an illness with COVID-19 be for you personally? Please use a scale from 0 to 10. A value of 0 means not at all severe and a value of 10 means very severe. You can also use one of the values in between to indicate your rating.”). Numerical risk perception was measured with one item employing a frequency format (Please imagine 1,000 adults like yourself. How many of these adults will contract COVID-19 as a result of close contact with someone infected with the virus?): Scoring of correct responses (5–28%) considered the range of the best available evidence, which can be found in S4 Table together with the medical references. For control, all items were presented with numbers for influenza as well, but were not the subject of this study.

Vaccination intention. Participants were asked if they planned to get vaccinated within the next six months.

#### Analysis

Besides descriptive statistics, repeated measures analyses of variance were conducted to compare the presentation conditions.

### Results

Studying the fact boxes decreased (Table 1) numeric COVID-19 risk perception (ANOVA interaction effect: *F*(1,357) = 10.05, *p =* .002, *n*_p_^2^ = 0.03) compared with control presentation, leading to more accurate estimates (see S3 Fig, *p* < .001). Only control presentation increased (Table 1) both personal fear (ANOVA interaction effect of time and presentation format, with *F*(1,357) = 4.17, *p =* .042, *n*_p_^2^ = 0.01) and perceived severity of developing COVID-19 (interaction, with *F*(1,357) = 19.90, *P* < .001, *n*_p_^2^ = 0.05).

**Table 1:** Personal and social fear, subjective and numeric risk perception across conditions, diseases, pre- and post-presentation.

Presentation format	Disease	Personal fear		Social fear		Subjective risk perception		Numeric risk perception (out of 1,000)	
		Pre	Post	Pre	Post	Pre	Post	Pre	Post
		M (SD)	M (SD)	M (SD)	M (SD)	M (SD)	M (SD)	M (SD)	M (SD)
Vaccine fact box	COVID-19	5.8 (2.6)	6.0 (2.7)	6.1 (2.8)	6.2 (2.8)	6.3 (2.5)	6.4 (2.6)	327 (294)	248 (222)
	Influenza	3.4 (2.4)	3.5 (2.5)	3.6 (2.5)	3.7 (2.7)	3.6 (2.4)	3.7 (2.6)	257 (246)	190 (192)
Social framing box	COVID-19	5.5 (2.5)	5.7 (2.6)	6.3 (2.4)	6.4 (2.4)	5.9 (2.4)	6.0 (2.3)	345 (323)	259 (245)
	Influenza	2.8 (2.1)	2.9 (2.1)	3.1 (2.0)	3.3 (2.2)	3.6 (2.0)	3.4 (2.2)	288 (294)	220 (242)
Standard information	COVID-19	5.5 (2.5)	6.0 (2.5)	6.3 (2.4)	6.9 (2.4)	5.5 (2.5)	6.2 (2.4)	317 (323)	314 (281)
	Influenza	2.9 (2.0)	3.4 (2.4)	3.2 (2.1)	4.0 (2.4)	3.3 (2.2)	3.8 (2.5)	207 (220)	171 (190)

More positive evaluations (*F*(1,357) = 12.55, *p* < .001, *n*_p_^2^ = 0.03) and increasing intentions to get vaccinated (*F*(1,357) = 7.63, *p* = .006, *n*_p_^2^ = 0.02) were shown as ANOVA main effects, but there were no format-specific interaction effects (S8 Table). Here, control information may have been as effective as fact boxes, albeit by promoting fear and perceived severity.

### Discussion

Presenting the vaccination fact box pilot decreased numerical disease risk perception and promoted accurate risk perceptions, without affecting vaccination intentions. At the same time, perceived severity of the disease and fear of contracting or spreading COVID-19 were not stimulated by the fact box presentation. Prompting fear responses would have posed the risk of interfering with informed decision-making [40]. Findings supported the further development and implementation of the fact box format and appeared to confirm the assumed relationship between fact box presentation and vaccination evaluation and intention.

## Study 4

Based on the insights of Studies 1, 2, and 3, Study 4 examined whether different fact box formats are effective for enabling informed vaccination intentions in a population-wide sample. Undecideds and skeptics needed and desired information (Study 2) to weigh potential benefits and harms [7]; otherwise they were hesitant to get vaccinated [8]. Study 4 tested whether both complex and simple fact boxes–based on the implemented version–would improve vaccination knowledge and modify vaccination evaluation or intention.

### Materials and methods

The study was conducted according to the conditions described under Study 1. Despite having received ethical approvals for similar fact box studies before [23, 24], we sought and received approval for the study design on mRNA fact boxes (IRB of the Max Planck Institute for Human Development, A 2021–07). The study was not pre-registered.

#### Participants

Study 4 was based on the sample T_3_ (S1 Table), described under Study 1. We excluded respondents (n = 182, 6.1%) who spent more than fivefold the average time (> about 18 minutes) on reading a fact box or on completing a question, assuming that they had likely turned to other activities.

#### Design

The experiment had a mixed experimental design, with between-subjects variation (random assignment of participants to one of three presentation conditions) and with repeated assessments of vaccination evaluation within-subjects (pre- and post-presentation). The three presentation conditions were (1) simple fact box (n = 984) (see Fig 3 and S4 Fig), complex fact box (n = 974) (see S5 and S6 Figs), and (3) one group without information presentation (n = 991). The formats varied for separate age groups (18–59 years vs. 60 onward). Participants were not aware of the alternative conditions. The same introductory text was provided for each condition.

#### Procedure

The procedure for the surrounding survey was described under Study 1. In Study 4, the vaccine efficacy item pair was presented either together with fact boxes (complex or simple) or without a stimulus (control group). Knowledge recall and a second vaccination evaluation were prompted after intervention.

#### Measures

Outcomes of interest in Study 4 were vaccination intention, evaluation, and vaccination knowledge.

Vaccination evaluation was assessed with participants’ ratings of the benefit-harm ratio of the COVID-19 vaccination in question on a 11-point rating scale from 0 (harms clearly outweigh the benefits) to 10 (benefits clearly outweigh the harms).

Vaccination intention and knowledge were assessed as described under Study 1. After information presentation, five items with true-false statements tested participants’ recall of vaccination safety (fatigue, serious adverse events), uncertainty (later harm, facial paresis), and efficacy.

Study time (in seconds) was the time that participants took for responding to the vaccine efficacy item pair, while some of them were presented with the interventions.

#### Analyses

We used variance analyses of intervention effects on knowledge and comprehension, and additional (logistic) regression analyses to examine factors influencing knowledge of and the evaluation of the vaccination (age group-specific formats, fact box formats, education, and income).

### Results

First, compared with no treatment, the sum score of vaccination knowledge was higher after fact box presentations (ANOVA main effect, *F*(1,3101) = 36.58, *p* < .001, *n*_p_^2^ = 0.01). Respondents of both observed age groups (below 60 and 60 onward) recalled vaccine efficacy, safety, and related uncertainties differentially when presented with a fact box instead of nothing (S9 Table). Multiple logistic regressions including the factors presentation format and age group showed that respondents of both age groups, who were presented with the fact box instead of nothing, were more likely aware of the side effect of fatigue (Odds Ratio (OR) = 1.85 with a 95%-confidence interval (CI) [1.69, 2.01]). Compared to those without fact box, respondents with fact box presentation, aged 60 and above, more likely confirmed a potential risk of facial paresis (OR = 1.16 [1.04, 1.28]), and younger respondents more likely recalled vaccine efficacy (OR = 1.13 [1.02, 1.24]).

Finally, vaccine efficacy was (OR = 1.85 [1.53, 2.17], *p* < .001) more likely correctly inferred when a fact box was presented (logistic regression controlling for the level of education).

Second, logistic regressions (*χ^2^*(1) = 14.70, *p* < .001 and *χ^2^*(1) = 5.50, *p* = .020) revealed that fact boxes more likely prompted both any change (OR = 1.36 [1.20, 1.52], *p* < .001) and a positive change (OR = 1.25 [1.06, 1.44], *p* = .020) of the evaluation of the vaccination compared with no intervention. For a distinct consideration of the different formats: Whereas 18.6% of respondents without any intervention changed their evaluation of a COVID-19 vaccination positively, 20.3% did so if studying a complex fact box, and 24.2% if studying a simple fact box. At the same time, however, 14.5%, 19.8%, and 16.3%, respectively, evaluated the evaluation more negatively when asked a second time (at post-assessment). Compared with no intervention, presenting a simple fact box (*χ^2^*(1) = 9.52, *p* = .002) more likely led to a positive than to a non-positive change (OR = 1.40 [1.19, 1.62], *p* = .002) in the evaluation of the vaccination. This could not be confirmed for the complex box (*χ^2^*(1) = 0.90, *p* = .343).

The shift in vaccination evaluation after being presented simple fact boxes (+7.9%) could, to a substantial extent, be related to the skeptical and undecided respondents’ comprehension of the information presented. Those who drew correct inferences about vaccine efficacy after having seen simple fact boxes showed a positive change in evaluation, but not those who drew incorrect inferences (main effect of an univariate ANOVA, *F*(1,467) = 3.88, *p* < .050, *n*_p_^2^ = 0.01). Explorative sub-analyses highlighted that this effect is due to the younger skeptics and undecideds (*F*(1,387) = 5.65, *p =* .018, *n*_p_^2^ = 0.01), not to those aged 60 and above (*F*(1,76) = 0.04, *p =* .836). In contrast, younger skeptics’ and undecideds’ recall, with at least 80% of correct responses after information presentation, was not related to positive changes in evaluation (*F*(1,387) = 0.59, *p =* .445).

Taking into consideration participants with different levels of education, we compared simple and complex fact boxes with no treatment. Respondents with the lowest educational qualification (16.5% with no school degree or degree after 9 years of schooling) recalled less than those with higher qualifications (degree after 10 years, university entrance qualification) (ANOVA main effect of sum score, *F*(1,3086) = 11.44, *p =* .001, *n*_p_^2^ < 0.01). But no interaction indicated an education-related recall benefit over the control condition (*F*(2,3086) = 0.20, *p =* .820).

**Fig 4:**
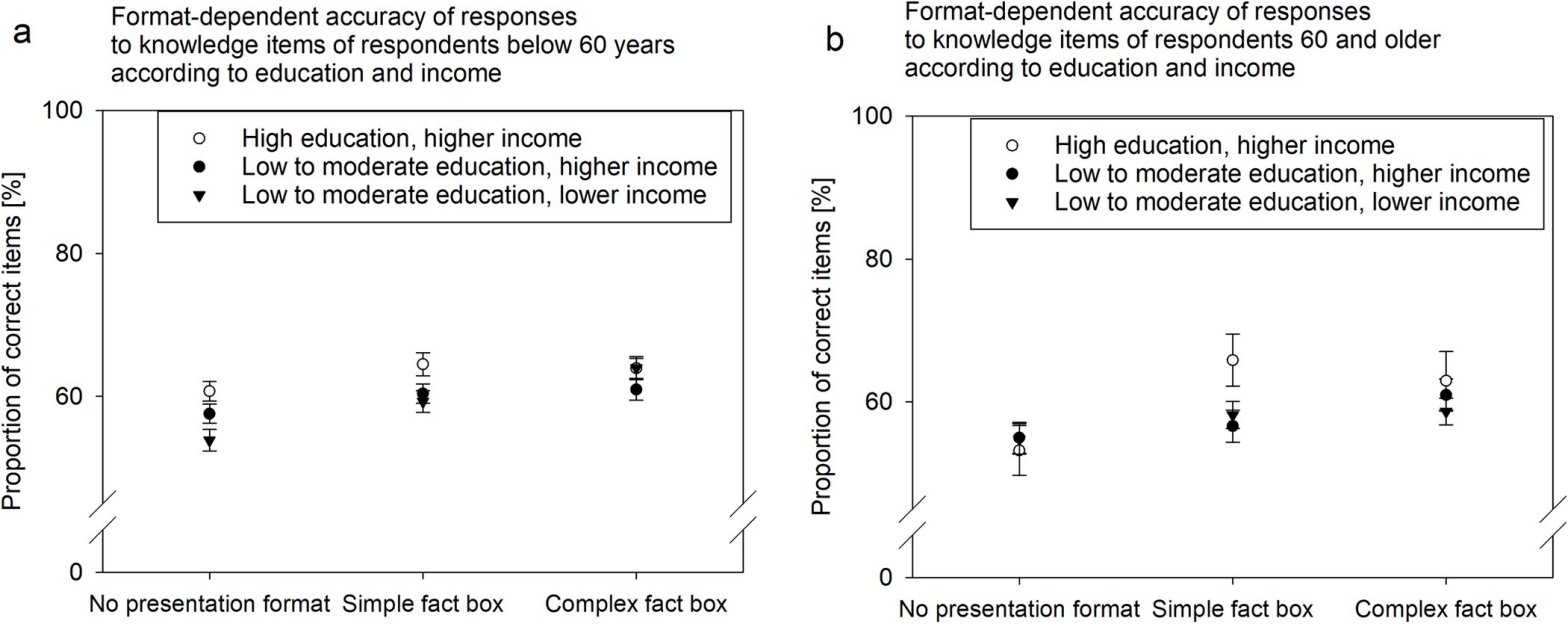
Proportion of correct responses to five knowledge items according to different levels of education and household net income for respondents younger than 60 years of age (A) and 60 years of age and older (B). The sample is weighted. Error bars show the standard error of the mean.

Additionally, we compared the two types of fact boxes. Recall of information from simple (59%) but not from complex fact boxes (61%) was significantly lower for respondents with low to moderate levels of education compared with those with higher levels (64% and 62%, respectively, ANOVA interaction effect, *F*(1,2048) = 4.10, *p =* .043, *n*_p_^2^ < 0.01). An additional analysis that split the low to moderately educated people according to their income median confirmed, across both age groups, that higher education, not income, was associated with recall of information from simple fact boxes (Fig 4A and 4B).

For complex fact boxes, the proportion of quick responders (below median response time of the control condition) among those with the lowest educational attainment was lower (7%; compared to those with higher education: 15%), which indicates that they needed more time for reading and/or for deciding to skip reading. In contrast, for simple fact boxes, the proportion of quick responders did not appear to depend on the level of education (in both groups about 11–12%).

### Discussion

People who read the fact boxes can be expected to gain a better comprehension of and short-term knowledge about the mRNA vaccination. Furthermore, their evaluation of the mRNA vaccination changes positively, particularly if those who were initially undecided and skeptical comprehend vaccine efficacy, e.g. via the simple fact box. A separate analysis could not confirm this net surplus for complex fact boxes. Furthermore, people with lower educational attainment seem to be less likely enabled to make an informed decision than higher educated groups, even when they receive this more detailed and complex version of the fact box. This indicates a potential risk of increasing health inequality by using evidence-based fact boxes, from which people with higher educational attainment benefit disproportionally.

## General discussion

The association of vaccination knowledge and uptake implies one crucial mechanism with respect to the goal of an adequately immunized society. Besides showing a differential increase in vaccination knowledge in proponents, undecideds, and opponents around the start of COVID-19 vaccination in Germany, Study 1 showed increasing intentions to have the (mRNA) vaccinations in the early phase of vaccine distribution [2]. Study 2 found that both undecideds and skeptics lacked various pieces of information for making a decision, paired with false beliefs and a lack of trust in vaccine safety and efficacy. Study 3 revealed that a COVID-19 vaccination fact box improved disease risk perception without increasing COVID-19 fears (unlike in the control condition). Based on these findings, a previously implemented vaccination fact box was amended. Applied at the population level in Study 4, fact boxes boosted knowledge of undecideds and skeptics together with a more positive evaluation of the vaccination’s benefit-harm ratio. Studying simple or complex fact boxes instead of nothing was 1.3 times more likely to lead to any positive change in evaluation. In the case of complex fact boxes, however, any changes seem to be balanced.

Although a common factor (e.g. peer behaviors) admittedly may underlie information acquisition and intentions–while stable vaccination preferences may prompt differential information acquisition–knowledge about benefits and harms of COVID-19 vaccinations, which can reduce vaccine hesitancy [41], may lead to informed intentions for readers of fact boxes if they base their intentions on these facts. This mechanism, in line with the standards of health care systems in many democratic countries, also contributes to the trustworthiness of authorities engaged in vaccination risk communication. For example, in face of the fact that denying information gaps can undermine perceived credibility [42, 43], fact boxes contain epistemic uncertainty disclaimers (e.g. verbal statements or ranges as a result of incomplete knowledge about the risk numbers) [18]. Transparently informing the public about limitations of vaccinations, which considers individual preferences, does not necessarily reduce vaccination intentions [44], which can support the societal preference to increase the vaccinating share of the population. By contrast, persuasive communications, e.g. messages framed in relative risks, can increase vaccination intentions [45] but are both misinformative and incomprehensible [46], colliding with the rights and needs of undecided and skeptical citizens. In addition, incomprehensible information may provoke a backlash effect. Future research could investigate the causal link between the grade of comprehensibility of information about benefits and harms and people’s vaccination intentions.

Limitations of our study: In our population-wide sample, simple fact boxes appear to be more beneficial for those with higher education. This contradiction to the design intention could be due to a superficial reading of the simple box by participants with lower levels of education, as indicated by their brief reading times. Although information needs and requirements were elicited, the target group with lower educational attainment did not actively participate in the development process of the fact box intervention (e.g., Study 3 included a highly educated convenience sample, separate piloting was missing). However, the potential of shared decision-making interventions to reduce health inequities [47] is tied to the involvement of disadvantaged groups in their development [48]. If neglected, there is a risk of increasing health inequity [49], e.g. due to the heterogeneity in the audience’s ability to comprehend given information. Those who comprehend the least, potentially benefit the least [45], e.g. low-numerate breast cancer patients [50]. Also, cancer screening fact boxes let high-numerate people update their benefit perceptions more strongly than low-numerates [22]. This could indicate distorted interpreting of fact boxes. Thus, more research needs to examine under which conditions fact boxes can strengthen informed decision-making of disadvantaged groups and reduce health inequities. Furthermore, concepts such as critical health literacy or digital health literacy should be taken into account to examine determinants that influence who benefits or does not benefit from fact boxes and how this affects health inequities [51]. Additional factors associated with formal education can also lead to inequality (e.g. working and living conditions). Future studies should thus incorporate approaches such as the PROGRESS Plus framework, which describes inequity-generating factors at multiple levels.

An additional limitation of our work concerns the set of vaccination knowledge items that covered certain requirements of health information guidelines (benefit, harms, uncertainty) but were not a validated scale of vaccination knowledge.

The results are hardly transferable to the non-democratic contexts, because, alongside the differences in media, political handling of the pandemic, and access to vaccination, the needs and preferences for the content and design of health information depend on a population’s risk perception, numerical literacy, and the opportunity for informed decision-making. This suggests that the success of a complex public health intervention, e.g. the provision of fact boxes by a central health authority, depends on a number of factors related to the societal characteristics of a country or region.

We conclude that in Germany, the implementation of fact boxes [52] supports evidence-based communication and, thus, empowerment at the population level. Dissemination of the mRNA fact boxes via the RKI media channels included their webpage with about 130 million visits in 2020, an RKI Twitter account, and the cellphone app of the Permanent Vaccination Commission in Germany. Our figures imply that presenting the about 11 million undecided and skeptical adults under 60 years of age in Germany with a simple fact box for about 90 seconds would familiarize more than 600,000 people with vaccine efficacy. A majority of them would evaluate vaccinations more positively. Also, by abstaining from persuasion, reluctance and distrust concerning the sender can be prevented or alleviated. Loss of trustworthiness is a relevant risk of alternative interventions that are nontransparent (e.g., nudges), persuasive (e.g., advertisement), or that exert pressure (e.g., for authorized access to public activities).

Finally, informed decision-making is not a threat to the goal of an adequately immunized population. To achieve this, ensuring the right of each individual to decide about their own health might be sufficient, while other types of social contracts [53] could be less important. The standard of evidence-based health care, legally binding in Germany [12] and benchmark of a democratic society, would ensure responsible vaccination decisions and future commitment when refreshments of the vaccination might be necessary or when individuals might have to decide to get vaccinated against other diseases.

## Supporting information

S1 Fig Overview of used data sets and study samples. *Data from the Waves 0 to 5 were not analyzed for this paper.
(TIF)

S2 Fig Experimental fact boxes from Study 3 about benefits and harms of the mRNA vaccine “In case of contact with coronavirus” (A), with social framing “If you spread coronavirus” (B).
(TIF)

S3 Fig Accuracy of COVID-19 risk perception (responding with a 5–28% probability of developing COVID-19 given close contact to an infected person; evidence from October 2020). Error bars show 95% confidence intervals.
(TIF)

S4 Fig English translation of the simple fact box for people 60 years and older from Study 4. (TIF)

S5 Fig English translation of the complex fact box for people between the ages of 18 and 60 from Study 4. (TIF)

S6 Fig English translation of the complex fact box for people 60 years and older from Study 4. (TIF)

S1 Table Descriptions of the quasi-representative samples (Studies 1, 2, and 4) underlying T_0_, T_1_, T_2_, T_3_, and T_4_. The data are not weighted.
(DOCX)

S2 Table Sample descriptions for Study 3, according to fact box conditions vs. control condition and across the study conditions). (DOCX)

S3 Table Responses to open questions of undecideds (pro and contra vaccination), skeptics, and opponents (contra only). (DOCX)

S4 Table Intention to get vaccinated across presentation formats and pre- and post-assessment in Study 3. (DOCX)

S5 Table Proportion of correct responses to five knowledge items (true-false statements) after information presentation (recall). (DOCX)

S6 Table Reasons for and against COVID-19 vaccination based on multiple-choice items (Study 2) that were presented depending on prior vaccination intentions. The data are weighted.
(DOCX)

S7 Table Items, plus best available evidence in December 2020 for the studies 1, 2, and 4. (DOCX)

S8 Table Best available evidence in October 2020 on transmissibility in a close-contact setting and manifestation rate given an infection of SARS-CoV-2 and influenza virus, focusing on the age groups 20–39, 40–59, and 60–79 years. Min and max represent a range that includes dark figures, vaccine efficacy, and conflicting study estimates.
(DOCX)

S9 Table Category system about information requirements (knowledge gaps) and (subjective) needs of vaccination proponents, undecideds, skeptics, and opponents. (DOCX)

S10 Table Sample descriptions for Study 4, according to simple vs complex fact box conditions vs. control condition and across the study conditions. The data are not weighted.
(DOCX)

S1 File Data file of the quasi-representative sample underlying T_0_. (SAV)

S2 File Data file of the quasi-representative sample underlying T_1_. (SAV)

S3 File Data file of the quasi-representative sample underlying T_2_. (SAV)

S4 File Data file of the quasi-representative sample underlying T_3_. (SAV)

S5 File Data file of the quasi-representative sample underlying T_4_. (SAV)

S6 File Syntax file of the analyses of Study 1. (SPS)

S7 File Syntax file of the analyses of Study 3. (SPS)

S8 File Syntax file of the analyses of Study 4. (SPS)

S9 File Stimulus of the control condition in Study 3. (PDF)

S10 File Data file of the convenience sample of Study 3. (SAV)

S11 File Intention data file including samples underlying T_0_ to T_4_. (SAV)

S12 File Knowledge data file combining samples underlying T_0_ to T_4_. (SAV)
