# Supplementary figures and images for: Fact boxes that inform individual decisions may contribute to a more positive evaluation of COVID-19 vaccinations at the population level
Source: PLoS One. 2022 Sep 12;17(9):e0274186. doi: 10.1371/journal.pone.0274186 (PMC9467356; doi:10.1371/journal.pone.0274186)

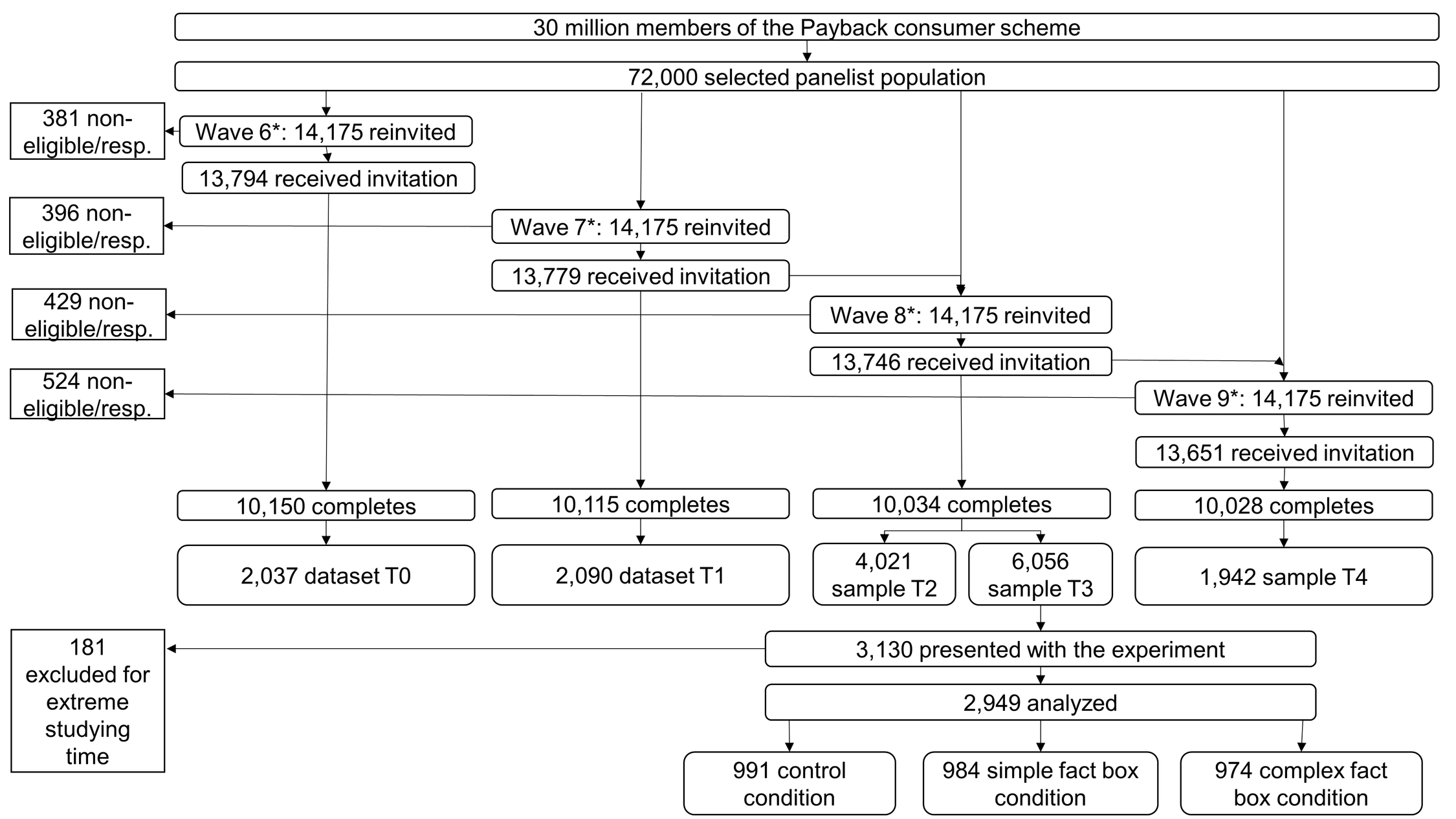

Supplement: S1 Fig — *Data from the Waves 0 to 5 were not analyzed for this paper. (TIF) [file pone.0274186.s001.tif]

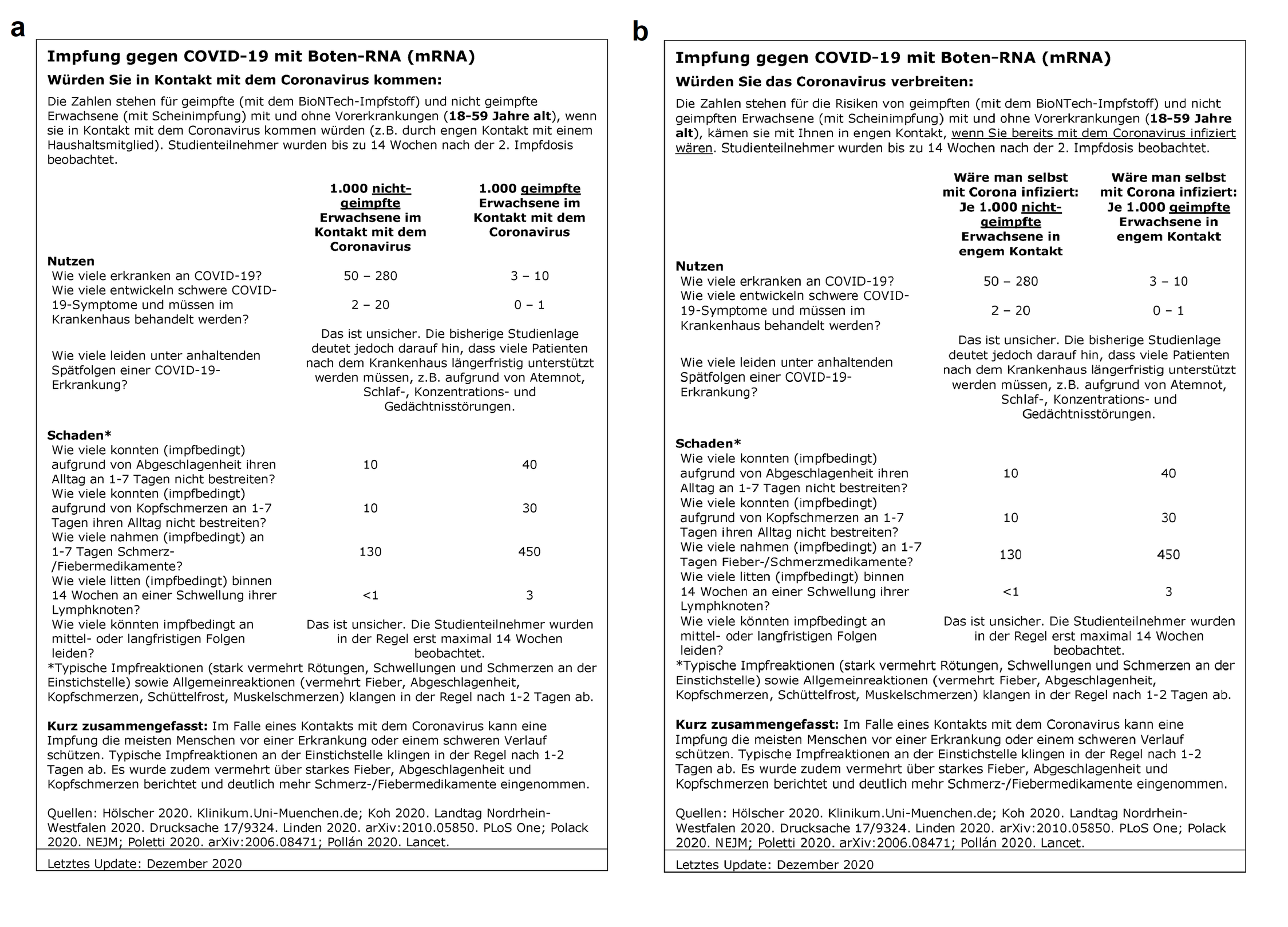

Supplement: S2 Fig — Experimental fact boxes from Study 3 about benefits and harms of the mRNA vaccine “In case of contact with coronavirus” (A), with social framing “If you spread coronavirus” (B). (TIF) [file pone.0274186.s002.tif]

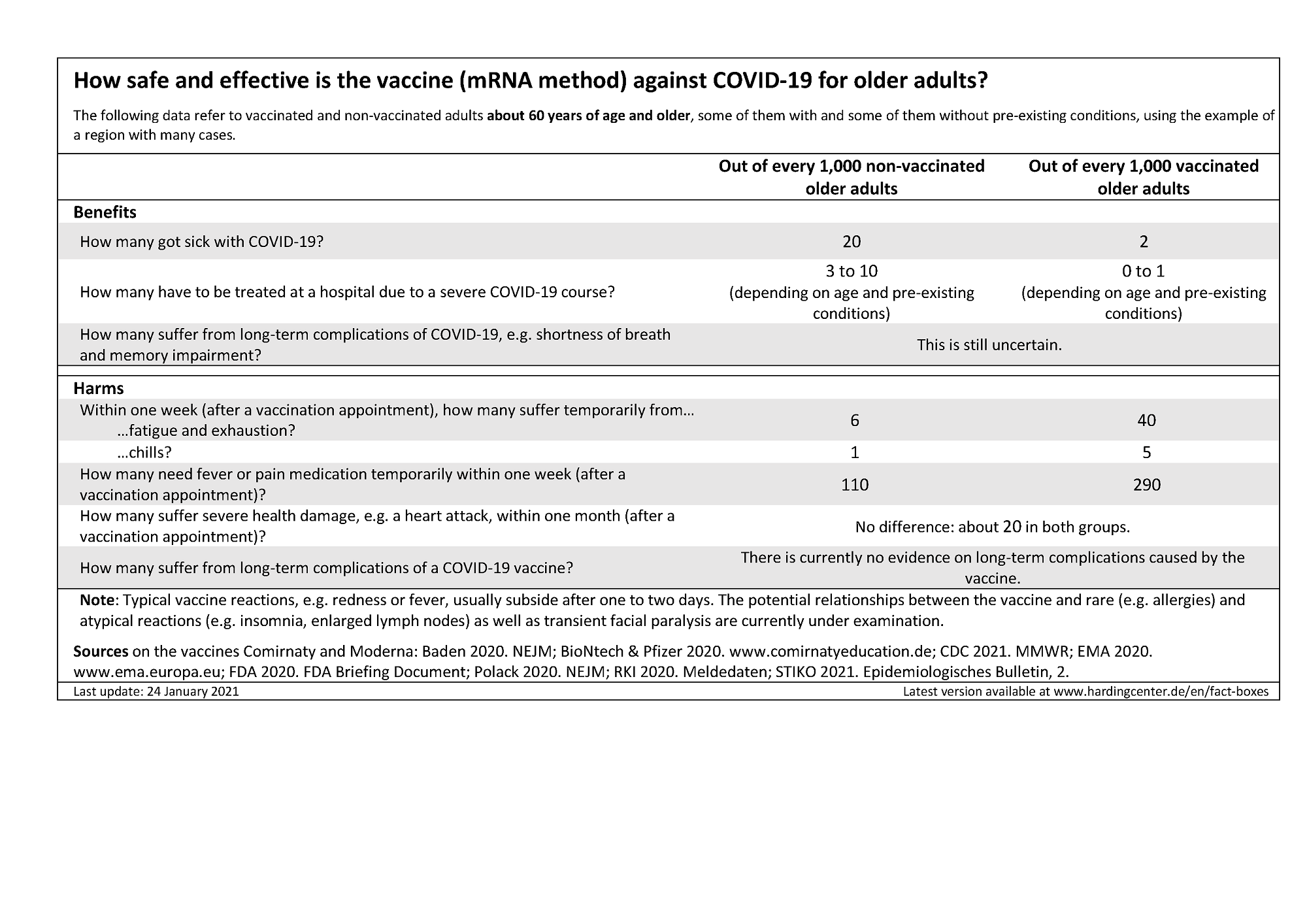

Supplement: S4 Fig — (TIF) [file pone.0274186.s004.tif]

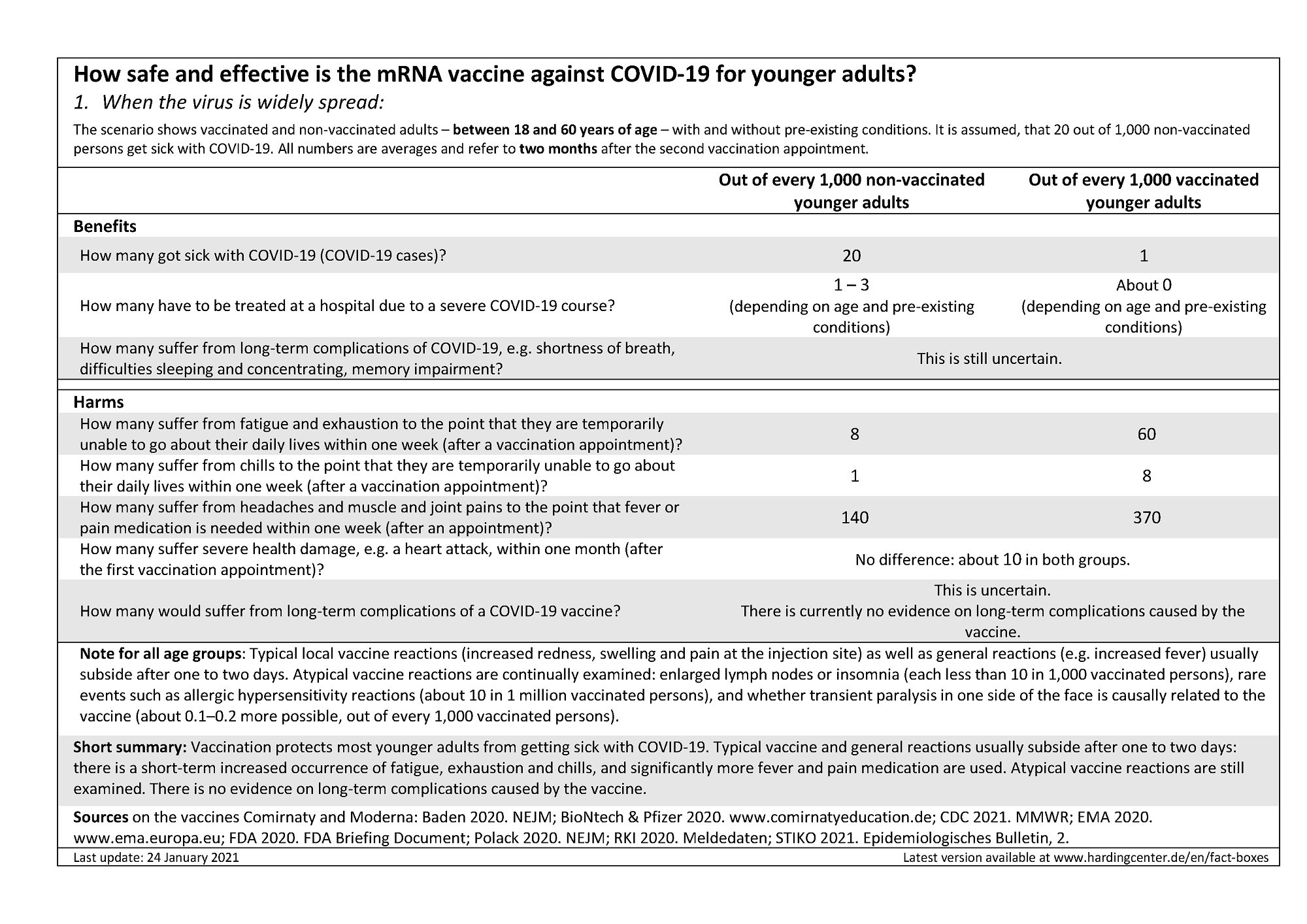

Supplement: S5 Fig — (TIF) [file pone.0274186.s005.tif]

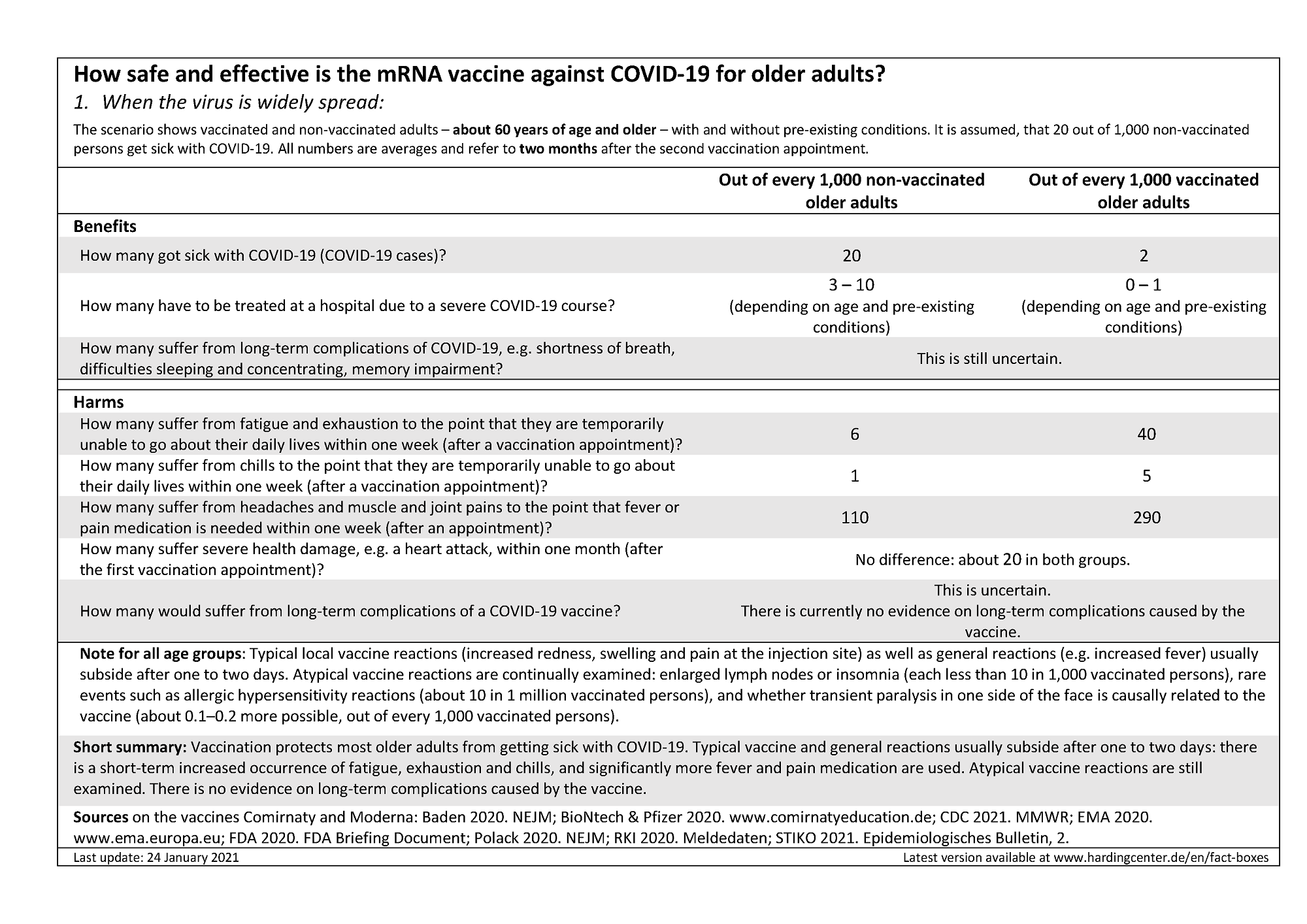

Supplement: S6 Fig — (TIF) [file pone.0274186.s006.tif]
