# Supplementary material for: Fact boxes that inform individual decisions may contribute to a more positive evaluation of COVID-19 vaccinations at the population level
Source: PLoS One. 2022 Sep 12;17(9):e0274186. doi: 10.1371/journal.pone.0274186 (PMC9467356; doi:10.1371/journal.pone.0274186)
Supplement: S1 Table — The data are not weighted. (DOCX) [file pone.0274186.s007.docx]

| Characteristic | Samples | T_0_ | T_1_ | T_2_ | T_3_ | T_4_ |
| --- | --- | --- | --- | --- | --- | --- |
|  | Studies | Studies 1 and 2 | Study 1 | Study 1 | Study 1 and 4 | Study 1 |
|  | COMPASS wave | 6 | 7 | 8 | 8 | 9 |
|  | Timespan | 25 Nov to 1 Dec 2020 | 28 Dec 2020 to 3 Jan 2021 | 6 Jan to 19 Jan 2021 | 20 Jan to 9 Feb 2021 | 10 to 16 Feb 2021 |
|  | N | 2,037 | 2,090 | 4,021 | 6,056 (2,949/Study 4) | 1,942 |
| Gender [%] | Female | 50.5 | 50.4 | 50.4 | 50.4 | 50.4 |
|  | Male | 49.5 | 49.6 | 49.6 | 49.6 | 49.6 |
| Age [M (SD)] |  | 52.1 (15.5) | 47.4 (15.3) | 47.3 (15.4) | 47.2 (15.4) | 47.2 (15.4) |
| Educational attainment (highest degree) [%] | No school degree  Primary school / 9 y  Secondary school / 10 y  Abitur / equivalent  Still in school education | 0.6  19.0  48.4  31.9  0.0 | 0.2  16.4  46.5  36.8  0.0 | 0.9  14.9  47.2  36.8  0.1 | 0.4  14.7  47.7  36.8  0.3 | 0.2  16.1  46.5  36.8  0.4 |
| Household net income [%] | 0 – 499 euros  500 - 999 euros  1,000 – 1,499 euros  1,500 – 1,999 euros  2,000 – 2,499 euros  2,500 – 2,999 euros  3,000 – 3,499 euros  3,500 – 3,999 euros  4,000 – 4,499 euros  4,500 – 4,999 euros  5,000 euros and more  Not specified | 1.3  4.6  7.5  11.0  13.1  10.9  9.1  7.7  4.9  3.7  7.0  19.2 | 0.8  4.4  7.6  10.0  12.5  13.1  9.8  8.2  5.6  4.0  7.5  16.6 | 1.3  4.3  7.8  11.9  11.1  11.6  8.7  7.3  5.6  4.7  7.3  18.4 | 1.3  3.8  7.4  10.5  11.4  11.5  10.3  8.5  6.1  4.5  8.0  16.8 | -  -  -  -  -  -  -  -  -  -  -  - |
| Number of inhabitants (place of living) | 1 – 1,999  2,000 – 4,999  5,000 – 19,999  20,000 – 49,999  50,000 – 99,999  100,000 – 499,999  500,000 and more | 2.9  8.5  23.4  18.5  10.0  17.3  19.5 | 4.7  9.1  27.8  17.5  10.5  15.2  15.2 | 4.1  8.9  27.4  19.4  9.4  14.9  15.9 | 4.1  9.3  27.4  19.5  9.0  14.7  16.0 | 4.2  8.1  28.3  19.1  9.1  15.0  16.2 |
| Respondents’ study device [%] | Desktop / laptop  Smartphone  Tablet / phablet | -  -  - | -  -  - | -  -  - | 49.5  44.6  5.9 | -  -  - |
