# Supplementary material for: Fact boxes that inform individual decisions may contribute to a more positive evaluation of COVID-19 vaccinations at the population level
Source: PLoS One. 2022 Sep 12;17(9):e0274186. doi: 10.1371/journal.pone.0274186 (PMC9467356; doi:10.1371/journal.pone.0274186)
Supplement: S2 Table — (DOCX) [file pone.0274186.s008.docx]

| Characteristic | Fact box conditions | Control condition | *p** | Total |
| --- | --- | --- | --- | --- |
|  | n=243 | n=116 |  | N=359 |
| Gender | 44.0% female  54.7% male  1.2% diverse | 28.4% female  71.6% male  0.0% diverse | .007^+^ | 39.0% female  60.2% male  0.8% diverse |
| Age | M=29.0,  SD=9.2 | M=28.3,  SD=8.3 | .733 | M=28.8,  SD=8.9 |
| Education | 74.5%  52.3% | 78.4%  56.0% | .413 | 75.8% university access qualification  53.5% university degree |
| Subjective socioeconomic status | 22.6%  38.7%  37.5% | 25.0%  33.6%  40.5% | .796 | 23.4% high  37.0% moderate  38.4% low |
| Predominant type of houses in the living area | 40.5%  59.5% | 37.0%  63.0% | .526 | 38.2% one-/two-family houses  61.8% apartment buildings |
| Familial real estate property | 64.2% | 66.4% | .685 | 64.9% yes |

* We conducted χ^2^-tests given categorical data, and a Mann-Whitney-U test for the variable age.

+ In the light of the technical randomization at the hosting site and the balanced variables we assume that gender varied by chance across conditions.
