# Supplementary material for: Fact boxes that inform individual decisions may contribute to a more positive evaluation of COVID-19 vaccinations at the population level
Source: PLoS One. 2022 Sep 12;17(9):e0274186. doi: 10.1371/journal.pone.0274186 (PMC9467356; doi:10.1371/journal.pone.0274186)
Supplement: S3 Table — (DOCX) [file pone.0274186.s009.docx]

| Item | Item options | Basic fact | Study |
| --- | --- | --- | --- |
| Vaccines against the Corona virusCOVID-19 are now available. If you have the opportunity, will you get vaccinated against Corona? | Definitely yes  Probably yes  Probably not  Definitely not  Undecided / I don’t know yet  (I already had the vaccination) | n.a. | 1, 4 |
| What are the reasons that speak for/against having the vaccination? | _________ | n.a. | 2 |
| Why do you not want to be vaccinated if necessary? | The pandemic will pass without causing any major damage. | n.a. | 2 |
|  | I think it is very unlikely that I will get infected | n.a. | 2 |
|  | Even if I get infected, I do not think that the disease will be really severe. | n.a. | 2 |
|  | I am still unsure about the side effects. | n.a. | 2 |
|  | I was already infected and I assume that I am immune. | n.a. | 2 |
|  | Other, namely: ___________ [open text box] | n.a. | 2 |
| [Baseline and post-assessment]  How do you personally assess the ratio of possible benefit and risk of the vaccination? | Please use a scale from 0 to 10. A value of 0 means that the risk clearly outweighs the possible benefit. A value of 10 means that the potential benefit clearly outweighs the risk. You can use the values in between to grade your assessment. | n.a. | 4 |
| To help you evaluate the vaccination, we provide informational materials below. Please read through the materials and use them to answer the following questions. | | n.a. | 4 |
| Please imagine 1,000 people who have (not) been vaccinated.  How many do you think would have developed COVID-19 if they had come into close contact with an infected person?  _______ People | 18 to 64 years | Risk ratio 0.04 | 1, 4 |
|  | 65 years + | Risk ratio 0.10 | 1, 4 |
| Please imagine 1,000 people who have (not) been vaccinated against COVID-19.  How many of them do you think are currently unable to carry out their everyday activities in a week because of headaches? | 18 to 64 years | Risk ratio 2.69 | 1 |
|  | 65 years + | Risk ratio 2.44 | 1 |
| Which of the following statements do you think is correct?  If people become seriously ill or die shortly after vaccination, then... | .  ..the vaccination is the cause | Incorrect | 1 |
|  | ...these people would have become seriously ill or died even without vaccination. | Unknown | 1 |
|  | ...these cannot be consequences of the vaccination because such consequences can already be ruled out. | Unknown | 1 |
|  | ...this is statistically recorded and officially evaluated throughout Germany. | Correct | 1 |
| What uncertainties still exist with regard to vaccination? | - The so-called messenger RNA used in the vaccine could change the genetic make-up of humans. | Incorrect | 1 |
|  | - The immune system could attack all cells in the body in response to the vaccine. | Incorrect | 1 |
|  | - The nanoparticles used in the vaccine, which enable the transport of messenger RNA in the body, could damage the nervous system. | Incorrect | 1 |
|  | - Vaccinated people could continue to infect other people with the virus. | Correct | 1 |
| In your opinion, are the following statements correct or incorrect?  If a younger person (under 60) receives a dose of the mRNA vaccine against COVID-19… | ...the risk is multiplied that one is too exhausted for everyday activities in the days afterward. | Correct | 4 |
|  | ...there is a 1% [2%] risk (10 [20] out of 1,000) of suffering serious health damage due to the vaccination. | Incorrect | 4 |
|  | ...it is uncertain whether the vaccination will have late effects. | Correct | 4 |
|  | ...it is certain that it does not lead to paralysis of the face. | Incorrect | 4 |
|  | ...the risk of getting severely ill with COVID-19 in contact with the coronavirus is reduced by a factor of about 20 [10].  Correct  Incorrect  I don’t know | Correct | 4 |
