# Supplementary material for: Fact boxes that inform individual decisions may contribute to a more positive evaluation of COVID-19 vaccinations at the population level
Source: PLoS One. 2022 Sep 12;17(9):e0274186. doi: 10.1371/journal.pone.0274186 (PMC9467356; doi:10.1371/journal.pone.0274186)
Supplement: S4 Table — (DOCX) [file pone.0274186.s010.docx]

|  | Influenza virus | | | SARS-CoV-2 | | |
| --- | --- | --- | --- | --- | --- | --- |
| Age 20 - 39 years | Min |  | Max | Min |  | Max |
| Secondary household attack rate (infection risk given close contact to index patient) | 0.05 |  | 0.19 | 0.24 |  | 0.42 |
| Manifestation rate of clinical symptoms | 0.15 |  | 0.35 | 0.22 |  | 0.62 |
| Vaccine efficacy | 0.40 |  | 0.00 |  |  |  |
| Out of 1,000 close contacts with infected people (equals our scoring benchmark) | 3 |  | 67 | 55 |  | 261 |
| Age 40 - 59 years |  |  |  |  |  |  |
| Secondary household attack rate (infection risk given close contact to index patient) | 0.05 |  | 0.19 | 0.24 |  | 0.42 |
| Manifestation rate of clinical symptoms | 0.15 |  | 0.35 | 0.31 |  | 0.64 |
| Vaccine efficacy | 0.40 |  | 0.00 |  |  |  |
| Out of 1,000 close contacts with infected people (equals our scoring benchmark) | 3 |  | 67 | 75 |  | 275 |
| Age: 60 - 79 years |  |  |  |  |  |  |
| Secondary household attack rate (infection risk given close contact to index patient) | 0.05 |  | 0.19 | 0.24 |  | 0.42 |
| Manifestation rate of clinical symptoms | 0.15 |  | 0.35 | 0.65 |  | 0.52 |
| Vaccine efficacy | 0.40 |  | 0.00 |  |  |  |
| Out of 1,000 close contacts with infected people (equals our scoring benchmark) | 3 |  | 67 | 158 |  | 219 |
| References:   - Demichelli et al. (2018). Vaccines for preventing influenza in healthy adults. Cochrane Database Sys. Rev.; - Gordon (2018). Influenza Transmission Dynamics in Urban Households, Managua, Nicaragua, 2012–2014. Emerg. Infect. Dis.; Leung (2015). Epidemiology, 26(6), 862-872; - Hölscher et al. (2020). Prospektive COVID-19 Kohorte München (KoCo19): Zusammenfassung der epidemiologischen Ergebnisse der Erstuntersuchung. - Koh et al. (2020). What do we know about SARS-CoV-2 transmission? A systematic review and metaanalysis of the secondary attack rate and associated risk factors. PLoS One; - Leung (2015). The fraction of influenza virus infections that are asymptomatic: a systematic review and meta-analysis. Epidemiology, 26(6), 862-872; - Linden et al. (2020). The foreshadow of a second wave: An analysis of current COVID-19 fatalities in Germany arXiv preprint arXiv:2010.05850. - Poletti et al. (2020). Probability of symptoms and critical disease after SARSCoV-2 infection. arXiv preprint arXiv:2006.08471. - Pollán et al. (2020). Prevalence of SARS-CoV-2 in Spain (ENE-COVID): a nationwide, population-based seroepidemiological study, Lancet; - RKI (2019). Bericht zur Epidemiologie der Influenza in Deutschland 2018/19; - WHO (2009). Weekly Epidemiol Rec; 84(25):249–257; | | | | | | |
