# Supplementary material for: Fact boxes that inform individual decisions may contribute to a more positive evaluation of COVID-19 vaccinations at the population level
Source: PLoS One. 2022 Sep 12;17(9):e0274186. doi: 10.1371/journal.pone.0274186 (PMC9467356; doi:10.1371/journal.pone.0274186)
Supplement: S5 Table — (DOCX) [file pone.0274186.s011.docx]

| Vaccination intention | Motivations of proponents | | Reasons for being undecided | | Reasons against vaccination given by skeptics and opponents | |
| --- | --- | --- | --- | --- | --- | --- |
|  | Code within category | Examples of proponents | Code within category | Examples of undecideds | Code within category | Examples of opponents |
| Confidence | More knowledge/research on safety (side effects, etc.) and efficacy (how effective is the vaccine?) | "More experience"  "Long-term testing"  "Research on the vaccine regarding (long-term) side effects" | Lack of research/uncertainty regarding efficacy and safety | "No research on possible long-term damage"  "There is no experience yet"  "Not knowing how the vaccine will interact with other medications. No knowledge about long-term effects" | Lack of research/uncertainty regarding efficacy and safety | "The vaccine has not been properly tested, so no one knows if there is vaccine-related damage"  "The vaccine can't have been adequately tested in such a short time."  "The vaccine needs to be tested for years" |
|  | No risk/absence of harm (no side effects, late effects, long-term effects) | "No side effects"  "When there is a perfectly tested, risk-free, and permanently available vaccine [...]"  "With 100% safety" | Fear about harms (disease caused by vaccination, side effects, late effects, long-term damage) | "Disease after vaccination, possible consequential damage due to immature vaccine"  "I am afraid of side effects"  "[...] The fear that one will get sick with corona afterwards. As it was often the case after flu vaccinations" | Fear about harm (disease caused by vaccination, side effects, late effects, long-term damage) | "Fear of side effects"  "If pregnancy is planned, fear of late effects, side effect for child"  "Fear of long-term damage caused by vaccination"  "Fear of side effects and unforeseeable long-term consequences" |
|  | High/long vaccine efficacy | "[...] the time the efficacy lasts is at least 1 year"  "[...] high effectiveness"  "If it is guaranteed that the vaccination makes people immune to the virus"  "To be immune to Corona."  "80% - 100% guarantee that it helps" | - |  | - |  |
|  | No confidence in/skepticism towards politics/efficacy | "Trust in politicians or virologists (Currently no trust and common sense is missing)"  "Very, very, very many convincing arguments"  "[...] more transparency and openness" | No confidence in/skepticism towards politics/efficacy | "Questionable if it will do any good"  "Confidence"  "No 100% certainty"  "It all happened so fast, how safe is it?" | No confidence in/skepticism towards politics/efficacy | "A vaccine cannot be adequately tested in such a short time, it usually takes 10 years"  "The vaccine only helps with old viruses and not with new ones."  "I'm not a guinea pig after all..."  "Vaccines are poisons used by the government to calm the population."  "[…] And I‘ll decide about this myself and certainly none of our stupid politicians!" |
| Extrinsic motives | Vaccination bonus | “Vaccination bonus” | - |  | - |  |
|  | Mandatory vaccination (general, by the employer) | "[...] Mandatory/recommended by the employer" | - |  | - |  |
|  | Physician' s /employer's recommendation, good experience of others | "[...] Mandatory/recommended by the employer"  "On doctor's advice" | - |  | - |  |
|  | More freedom, to experience no more restrictions or no disadvantages (reversed) | "To be able to have all freedoms again without restriction"  "To be allowed to travel"  "If it becomes obligatory for traveling abroad, for example"  "Fewer contact restrictions" | - |  | - |  |
| Intrinsic motives | Self-protection (I can protect myself from the disease) | "To avoid getting infected"  "My health, [...]"  "My cancer" | Personal reasons against vaccination | "Not healthy"  "Personal reasons, always got incredibly sick even after the flu shot"  "I have a lot of allergies to medications and also herbal agents. Likewise, I have a previously unknown allergy, I sneeze all day, sometimes up to 15 minutes in a row. Causes can not be clarified. [...]"  "My pregnancy"  "I don't want to be a guinea pig because of my illness" | Personal reasons against vaccination | "I know of vaccination damage"  "Can't even tolerate the flu vaccine"  "Pregnancy” |
|  | Personal responsibility (I can decide for myself whether I want to be vaccinated) | "Decision is made at own discretion"  "Voluntariness" | Personal responsibility (I can decide for myself whether I want to be vaccinated) | "None at all. Let everyone decide for themselves whether and how they assess the risk."  "None so far, actually" | Personal responsibility (I can decide for myself whether I want to be vaccinated) | "I'm against vaccination in general!"  "Because I don't want it"  "I won't be injected with anything that has been so poorly researched. I have only one life. And I‘ll decide about this myself and certainly none of our stupid politicians!"  "Vaccination opponents" |
| Complacency  (risk perception) | Increase in the risk of disease | "More risk of infection, overburdening of the health care system"  "When infection increases"  "Diseases in the closer environment" | Low overall COVID-19 risk (No risk from COVID-19) | “Number of deaths”  "My attitude towards Corona"  "If it were really very bad" | Low overall COVID-19 risk (No risk from COVID-19) | "It is exaggerated (6 acquaintances had it and all were fine)"  "Socially important people and more affected countries should be vaccinated first"  "I think there will be many worse viruses and I don't want to get so many vaccinations. Maybe soon there will be a ‚nicer‘ virus where it is more important to get vaccinated"  “Panic” |
|  | No fear of COVID-19 disease, no need for vaccination | "Nothing"  "Nothing at all" | No fear of COVID-19 disease, no need for vaccination | "Good immune system"  "[...] Not a high-risk patient"  "I consider myself sufficiently fit to get through it without a vaccination. I think that other people need it more and so I would put myself in the back of the queue for the time being. [...]".  "Am not in the medical/nursing sector and not really in a risk group"  "Am not active outside my home" | No fear of COVID-19 disease, no need for vaccination | "The people who need it should get vaccinated"  "I just hope it doesn't hit me"  "I've managed without a flu shot so far"  "I never get vaccinated" |
| Collective responsibility (sense of responsibility for the community) | Protection of others (family, patients, etc.) | "One wants to protect oneself and one's surroundings and help to ensure that we can soon live again without worries."  "My health, and also that of my family and the people I love/care about."  "Protection of others in my profession, [...]"  "Protection of relatives" | Protection of others (family, patients, etc.) | "To contain the pandemic"  "[...] In addition, the willingness to show consideration for others then decreases" | Protection of others (family, patients, etc.) | "Socially important people and more affected countries should be vaccinated first" |
| Constraints (barriers to implementation) | e.g. availability (too expensive, long travel distances) | " Would not get vaccinated until after pregnancy and lactation if at all" | e.g. availability (too expensive, long travel distances) | "Terrified of injections"  "Too expensive, [...]"  “When long travel distances exist” | e.g. availability (too expensive, long travel distances) | "I'm afraid of needles" |
| Calculation (extent of information search) | Need for more information on risks, vaccine side effects | "Reasonable reporting of the media , not only of those who tested positive, but also recovered and tested negative. So that one can really get the whole picture. Thereafter, one can also judge whether to be vaccinated or not"  "Sufficient information because of side effects"  "Enlightenment" | Need for more information on risks, vaccine side effects | "I don't know the risks exactly. I would like to know more details about vaccination"  "Not enough information at this time"  "Not enough info about side effects and not enough experience with long-term effects"  "I am not sufficiently informed." | Need for more information on risks, vaccine side effects | “Do not know what they inject me with”  “[…] No exact info on the vaccine” |
| Other | Still unclear | "I don't know yet"  "Don't know" | Still unclear | "Don't know" | - |  |
|  | No information, not usable, not assignable | “?”  “???”  “…” | No information, not usable, not assignable | “?”  “???”  “No specification” | No information, not usable, not assignable | “I will not vaccinate for now but I am not against it”  “Nobody talks about flu deaths anymore at the moment” |
