# Supplementary material for: Fact boxes that inform individual decisions may contribute to a more positive evaluation of COVID-19 vaccinations at the population level
Source: PLoS One. 2022 Sep 12;17(9):e0274186. doi: 10.1371/journal.pone.0274186 (PMC9467356; doi:10.1371/journal.pone.0274186)
Supplement: S6 Table — The data are weighted. (DOCX) [file pone.0274186.s012.docx]

| Reasons in favor of having the vaccine | Proponents (probably or definitely having the vaccination) | Arguments against having the vaccine | Skeptics and opponents probably or definitely not having the vaccination |
| --- | --- | --- | --- |
|  | [%] |  | [%] |
| To protect one’s own health | 95.4 | Pandemic will pass without larger harm | 20.0% |
| To protect one’s relatives | 97.5 | Unlikely to get infected | 10.1% |
| To contribute to society | 87.3 | Belief in no severe course of illness in case of infection | 36.2% |
| To support the economic growth | 79.1 | Immuno-protected due to past infection | 1.1% |
| To abolish COVID-19 measures | 90.6 |  |  |
| Professional contact with humans | 52.9 |  |  |
