# Supplementary material for: Fact boxes that inform individual decisions may contribute to a more positive evaluation of COVID-19 vaccinations at the population level
Source: PLoS One. 2022 Sep 12;17(9):e0274186. doi: 10.1371/journal.pone.0274186 (PMC9467356; doi:10.1371/journal.pone.0274186)
Supplement: S7 Table — (DOCX) [file pone.0274186.s013.docx]

| Category of arguments related to vaccination intention | Coded arguments within category (Examples) | Undecideds | | Skeptics and opponents |
| --- | --- | --- | --- | --- |
|  |  | Pro | Contra | |
|  |  | Proportion [%] | | |
|  |  | N=248 | N=248 | N=262 |
| Confidence | [All codes] | 56.9 | - | - |
|  | More knowledge/research on vaccine safety and efficacy | 35.9 | - | - |
|  | No risk/absence of harm (no side effects, late effects, long-term effects) | 13.7 | - | - |
|  | High/long-term vaccine efficacy | 7.3 | - | - |
|  | No confidence in/skepticism towards politics/vaccine effectiveness | 0.4 | - | - |
|  | [All codes] | - | 98.4 | 98.5 |
|  | Insufficient research/uncertainty regarding efficacy and safety ("Not knowing how the vaccine will interact with other medications. No knowledge about long-term effects") | - | 53.2 | 43.1 |
|  | Fear of harm, including illness caused by vaccination, side effects, late effects, or long-term consequences ("I am afraid of side effects") | - | 30.6 | 39.3 |
|  | No confidence in/skepticism towards politics/vaccine effectiveness (“No 100% certainty") | - | 14.5 | 16.0 |
| Extrinsic motives | [All codes] | 14.1 | - | - |
|  | More freedom, to experience no more restrictions or no disadvantages (reversed) | 6.5 | - | - |
|  | Physician's /employer's recommendation, good experience of others | 4.4 | - | - |
|  | Mandatory vaccination (general, by the employer) | 2.8 | - | - |
|  | Vaccination bonus | 0.4 | - | - |
| Intrinsic motives | [All codes] | 10.1 | 5.6 | 10.7 |
|  | Self-protection ("Personal reasons, always got incredibly sick even after the flu shot") | 7.7 | - | - |
|  | Personal reasons against vaccination | - | 5.2 | 8.0 |
|  | Personal responsibility ("None at all. Let everyone decide for themselves whether and how high they assess the risk.") | 2.4 | 0.4 | 2.7 |
| Complacency (risk perception) | [All codes] | 5.2 | 4.4 | 6.5 |
|  | No fear of COVID-19 disease, no need for vaccination ("I consider myself sufficiently fit to get through it without a vaccination. I think that other people need it more and so I would put myself in the back of the queue for the time being. [...]") | 3.2 | 2.4 | 4.6 |
|  | Increase in the risk of disease | 2.0 | - | - |
|  | Low COVID-19 risk (“Number of deaths”) | - | 2.0 | 1.9 |
| Collective Responsibility | Protection of others: family, patients, etc. ("To contain the pandemic") | 8.5 | 0.8 | 0.4 |
| Constraints (barriers to implementation) | Availability: too expensive, long traveling (“"When long travel distances are required") | 0.8 | 0.8 | 0.4 |
| Calculation (Extent of information search) | Need for more information on risks, e.g. vaccine side effects ("I don't know the risks exactly. I would like to know more details about vaccination") | 8.9 | 4.8 | 0.4 |
| Other | [All codes] | 12.1 | 9.3 | 0.4 |
|  | Still unclear | 7.7 | 3.2 | 0.0 |
|  | No information, not usable, not assignable | 4.4 | 1.6 | 0.4 |
|  | No reasons against | - | 4.4 | 0.0 |
