# Supplementary material for: Fact boxes that inform individual decisions may contribute to a more positive evaluation of COVID-19 vaccinations at the population level
Source: PLoS One. 2022 Sep 12;17(9):e0274186. doi: 10.1371/journal.pone.0274186 (PMC9467356; doi:10.1371/journal.pone.0274186)
Supplement: S8 Table — Min and max represent a range that includes dark figures, vaccine efficacy, and conflicting study estimates. (DOCX) [file pone.0274186.s014.docx]

| Intention to get vaccinated | Vaccine fact box | | Social framing box | | Standard information | |
| --- | --- | --- | --- | --- | --- | --- |
|  | Baseline  [%] | Post  [%] | Baseline  [%] | Post  [%] | Baseline  [%] | Post  [%] |
| Definitely not | 5.8 | 5.0 | 8.1 | 8.9 | 6.9 | 6.0 |
| Probably not | 18.3 | 15.0 | 15.4 | 10.6 | 16.4 | 12.9 |
| Undecided | 13.3 | 17.5 | 15.4 | 21.1 | 11.2 | 13.8 |
| Probably yes | 29.2 | 29.2 | 28.5 | 26.0 | 35.3 | 35.3 |
| Definitely yes | 33.3 | 33.3 | 32.5 | 33.3 | 30.2 | 31.9 |
