# Supplementary material for: Fact boxes that inform individual decisions may contribute to a more positive evaluation of COVID-19 vaccinations at the population level
Source: PLoS One. 2022 Sep 12;17(9):e0274186. doi: 10.1371/journal.pone.0274186 (PMC9467356; doi:10.1371/journal.pone.0274186)
Supplement: S9 Table — (DOCX) [file pone.0274186.s015.docx]

| Age group | Presentation format | Safety: fatigue | Safety: serious adverse event | Uncertainty: later harm | Uncertainty: facial paresis | Efficacy: risk factor |
| --- | --- | --- | --- | --- | --- | --- |
|  |  | [%] | [%] | [%] | [%] | [%] |
| Below 60 years | Without information presentation | 39.8 | 50.2 | 71.7 | 56.7 | 66.7 |
|  | Simple fact box | 54.0 | 50.4 | 72.9 | 60.1 | 68.1 |
|  | Complex fact box | 55.0 | 52.7 | 74.5 | 58.5 | 72.2 |
| 60+ | Without information presentation | 32.8 | 61.9 | 60.5 | 40.6 | 71.9 |
|  | Simple fact box | 48.0 | 58.1 | 67.3 | 43.8 | 76.1 |
|  | Complex fact box | 49.4 | 56.4 | 64.3 | 54.8 | 70.5 |
