# Supplementary material for: Fact boxes that inform individual decisions may contribute to a more positive evaluation of COVID-19 vaccinations at the population level
Source: PLoS One. 2022 Sep 12;17(9):e0274186. doi: 10.1371/journal.pone.0274186 (PMC9467356; doi:10.1371/journal.pone.0274186)
Supplement: S10 Table — The data are not weighted. (DOCX) [file pone.0274186.s016.docx]

| Characteristic | Samples | Simple fact box | Complex fact box | Control condition | *p** | Total |
| --- | --- | --- | --- | --- | --- | --- |
|  |  | n=984 | n=974 | n=991 |  | N=2,948 |
| Gender [%] | Female | 51.4 | 52.5 | 46.9 | .030 | 50.3 |
|  | Male | 48.6 | 47.5 | 53.1 |  | 49.7 |
| Age [M (SD)] |  | 47.9  (14.9) | 47.3  (15.4) | 46.2  (15.9) | .065 | 47.1 (15.4) |
| Educational attainment (highest degree) [%] | No school degree  Primary school / 9 y  Secondary school / 10 y  Abitur / equivalent  Still in school education | 0.2  16.8  48.2  34.1  0.7 | 0.5  14.7  47.5  36.6  0.7 | 0.8  14.3  44.9  40.1  0.0 | .012^+^ | 0.5  15.3  46.8  36.9  0.5 |
| Household net income [%] | 0 – 499 euros  500 - 999 euros  1,000 – 1,499 euros  1,500 – 1,999 euros  2,000 – 2,499 euros  2,500 – 2,999 euros  3,000 – 3,499 euros  3,500 – 3,999 euros  4,000 – 4,499 euros  4,500 – 4,999 euros  5,000 euros and more  Not specified | 1.0  4.2  7.5  8.6  11.3  13.2  12.3  7.5  4.4  4.8  8.6  16.7 | 1.4  3.5  6.5  12.7  11.4  11.0  10.7  8.5  6.5  5.5  5.9  16.3 | 1.4  3.7  7.3  10.9  12.3  12.5  8.6  8.0  6.7  3.2  8.7  16.6 | .023 | 1.3  3.8  7.1  10.7  11.7  12.3  10.5  8.0  5.9  4.5  7.8  16.6 |
| Respondents’ study device [%] | Desktop / laptop  Smartphone  Tablet / phablet | 50.5  44.0  5.5 | 47.6  45.8  6.6 | 50.0  44.2  5.8 | .682 | 49.4  44.7  5.9 |

* We conducted χ^2^-tests given categorical data, and a Kruskal-Wallis-H test for the variable age.
+ Potential influence of the factor education was analyzed separately (Results section of Study 4).
