# Supplementary material for: Fact boxes that inform individual decisions may contribute to a more positive evaluation of COVID-19 vaccinations at the population level
Source: PLoS One. 2022 Sep 12;17(9):e0274186. doi: 10.1371/journal.pone.0274186 (PMC9467356; doi:10.1371/journal.pone.0274186)
Supplement: S9 File — (PDF) [file pone.0274186.s025.pdf]

Reference: https://www.helios-gesundheit.de/magazin/corona/news/corona-versus-grippe-was-ist-gefaehrlicher/
(Last access of the study material, 12 December 2020)

Corona versus Grippe: Der Unterschied liegt im Detail

Die Zahl der Sars-CoV-2-Infizierten steigt weltweit. Eine Infektion mit dem Virus kann tödlich enden – die Infektion mit echten Grippeviren ebenso. Doch wie unterscheiden sich Grippe und Corona in Bezug auf Symptome, Verlauf und Therapie?

Corona (Covid-19) weist auffällig **viele Ähnlichkeiten zur Grippe** (Influenza) auf. So ist sie ähnlich ansteckend, ungefähr genauso tödlich und für dieselben Risikogruppen gefährlich. Und doch ist es damit nicht getan. Auch wenn die Grippe nicht harmlos ist, ist sie für den menschlichen Organismus nicht neu. Zwar stecken sich jedes Jahr viele Menschen daran an, aber die Sterblichkeit ist mit wenigen Zehntelprozent relativ gering.

Wie gefährlich eine **Infektion mit Sars-CoV-2** wirklich ist, ist noch nicht klar, denn das Corona Virus und die daraus resultierende Erkrankung Covid-19 sind noch nicht ausreichend erforscht. Die vorhandenen Daten lassen darauf schließen, dass der Krankheitsverlauf einer Influenza und von Covid-19 etwa gleich gefährlich sein kann. Vorausgesetzt, schwere Verläufe können medizinisch ideal behandelt werden.

Corona-Virus befällt nicht nur die unteren Atemwege

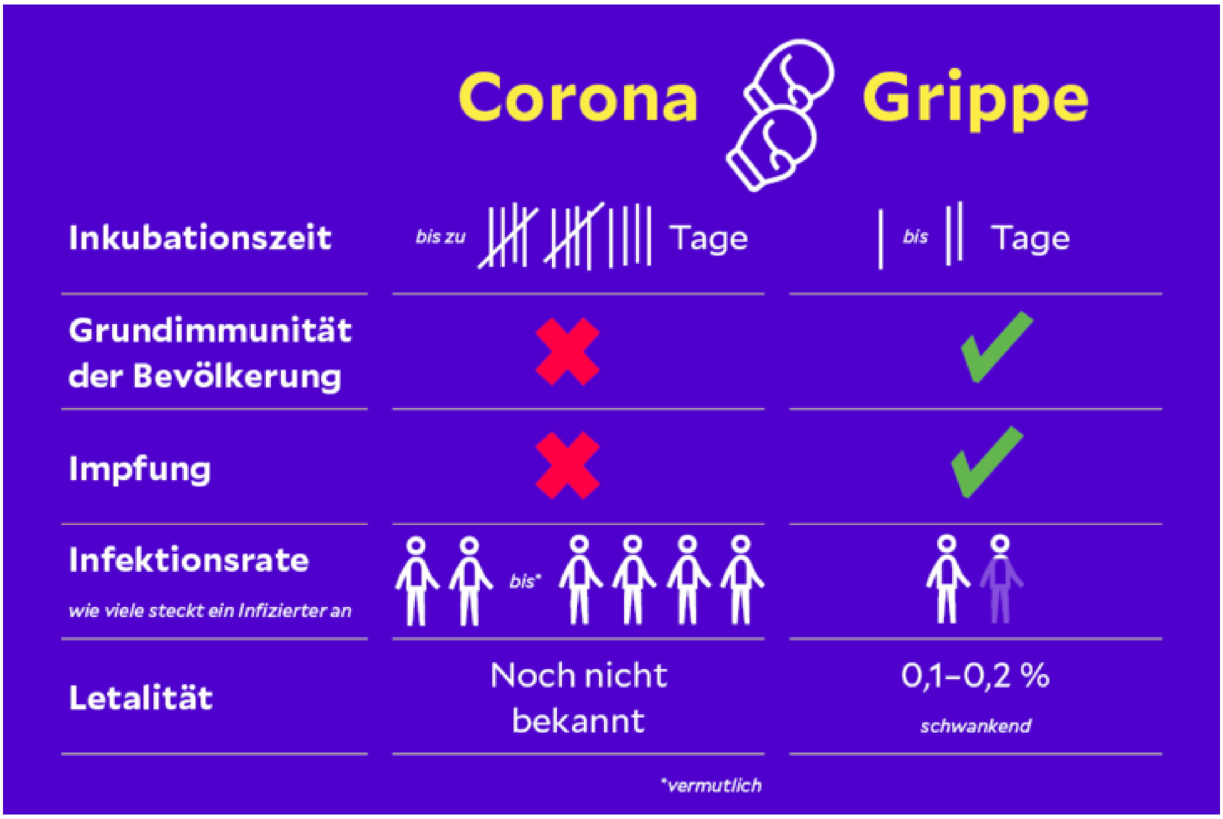

Ein Vergleich zwischen Covid-19 und Grippe | Grafik: Helios

Zunächst galt Covid-19 als reine Lungenerkrankung, die primär die unteren Atemwege betrifft. Mittlerweile ist klar: das Virus beeinflusst auch andere Organe und die Blutgerinnung.

Erhöhte Ansteckungsgefahr bei Corona

Influenza-Viren als auch Corona-Viren werden **durch Tröpfchen übertragen**, die Infizierte beim Sprechen, Husten oder Atmen ausstoßen.

Bei der Übertragung der beiden Viren, insbesondere bei Covid-19, spielen auch **Aerosole** (feinste Schwebetröpfchen) eine Rolle. Da sich Aerosole in der Luft anreichern können, besteht in geschlossenen Räumen ein erhöhtes Infektionsrisiko mit Sars-CoV-2.

Das Corona-Virus ist im Vergleich zum Grippevirus **aus zwei Gründen ansteckender**: Zum einen fehlt die Grundimmunität in der Bevölkerung und zum anderen ist die Inkubationszeit länger.

Fehlende Grundimmunität in der Bevölkerung

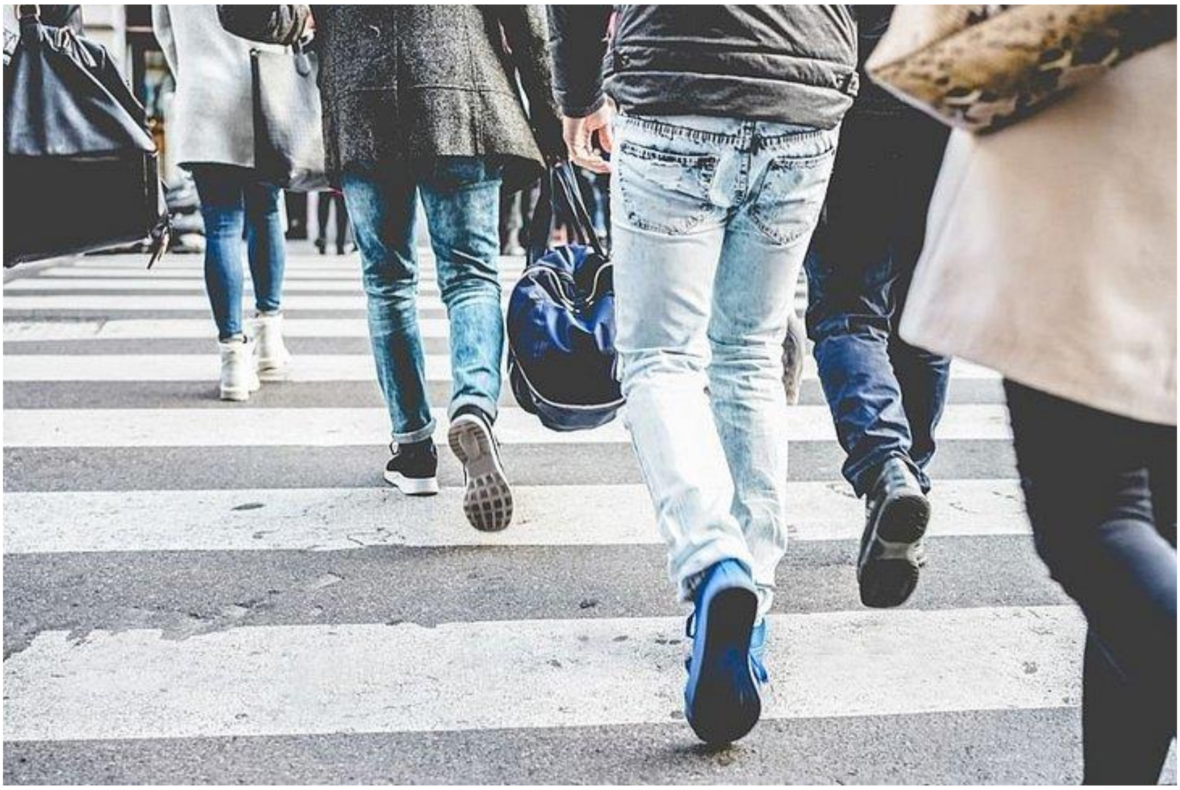

Gegen das Corona-Virus fehlt die Grundimmunität in der Bevölkerung | Foto: Canva

Die Gefahr von Covid-19 liegt darin, dass die Erkrankung neu aufgetaucht ist und neue, bis dahin unbekannte Oberflächenmerkmale aufweist. In der Bevölkerung fehlt die Grundimmunität gegen dieses Virus – und das ist einer der entscheidenden Unterschiede zur Grippe.

Ohne die Grundimmunität in der Bevölkerung ist es für den Erreger **leichter, sich zu verbreiten**. Die Wissenschaft geht zurzeit davon aus, dass eine gewisse **Kreuzreaktion zu den Erkältungs-Coronaviren** besteht und deswegen bei einem Teil der Bevölkerung eine **bedingte Immunität** vorliegt.

Mit dem Virus Sars-CoV-2 war bis zu seinem Ausbruch niemand infiziert. Wer sich jetzt ansteckt, kann erkranken und kann bei schwerem Verlauf zum Beispiel aufgrund einer Vorerkrankung an den Folgen der Infektion sterben. Wie viele Ansteckungen völlig symptomlos verlaufen wissen wir noch nicht, das lässt sich erst im Nachhinein sagen.

Inkubationszeit der Viren im Vergleich

Die Inkubationszeit liegt beim Corona-Virus bei **2 bis 14 Tagen**. Zum Vergleich: Bei einer Grippe liegt die Inkubationszeit bei 1 bis 2 Tagen. Somit können Menschen den Erreger bereits per Tröpfcheninfektion oder durch Aerosole übertragen, ohne zu wissen, dass sie schon erkrankt sind. Sie tragen den Erreger schon in sich, ohne es zu merken. Das ist ein entscheidender Unterschied zur Grippe.

Ähnliche Symptome bei Grippe und Corona

Laien können die Symptome einer Grippe meist schlecht von denen einer Covid-19-Erkrankung unterscheiden. Bei Verdacht auf Corona ist entscheidend, ob es **mögliche Kontakte zu Erkrankten** gibt oder gab.

Zu den **Hauptsymptomen von Covid-19** zählen Fieber, trockener Husten und Atemnot. Es kann zu Atemproblemen bis hin zu einer Lungenentzündung kommen. Typische Erkältungssymptome wie Schnupfen und Niesen treten im Vergleich seltener auf.

Zu den **Hauptsymptomen bei Grippe** zählen neben dem typischen trockenen Husten und plötzlich einsetzendem, oft hohem Fieber auch ein starkes Krankheitsgefühl sowie Kopf-, Muskel- und Gelenkschmerzen. Der Verlauf kann mild oder ernsthaft sein – wie bei Covid-19.

Den deutlichsten Unterschied zwischen Covid-19 und Grippe stellt die **Geruchs- und Geschmacksstörung** dar.

Symptome im Vergleich bei COVID-19 und Grippe

Symptome	COVID-19	Grippe
Fieber	*häufig*	*häufig*
Müdigkeit	manchmal	*häufig*
Husten	*häufig (trocken)*	*häufig (trocken)*
Niesen	nein	nein
Gliederschmerzen	manchmal	*häufig*
Schnupfen	selten	manchmal
Halsschmerzen	manchmal	manchmal
Durchfall	selten	manchmal (Kinder)
Kopfweh	manchmal	*häufig*
Kurzatmigkeit	manchmal	nein
Geschmacks- und Geruchsverlust	sehr häufig	nein

Therapie der Erkrankungen

Die Behandlung ist bei einer Covid-19-Infektion und bei einer Grippe Erkrankung ähnlich. Da es nur begrenzt Medikamente gibt, die gegen den Grippe-Virus wirken, werden primär die Symptome bekämpft und Körperfunktionen unterstützt. In ernsten Fällen etwa per künstlicher Beatmung und Unterstützung der Lungenfunktion durch spezielle Geräte (ECMO).

Corona Risikogruppen und –faktoren

Die Risikogruppe ist bei **beiden Erkrankungen ähnlich**. Covid-19 ist vornehmlich für Menschen mit Vorerkrankungen und Immunschwäche gefährlich und kann zu schweren Krankheitsverläufen führen.

Zu den **Risikogruppen** zählen neben älteren Menschen und Rauchern auch Menschen mit Vorerkrankungen wie:

- Herz-Kreislauf-Erkrankungen
- Diabetes
- Asthma
- Krebs
- Geschwächtes Immunsystem
- Chronische Lebererkrankung
- Übergewicht oder Adipositas (Fettleibigkeit)

Wie kann ich mich vor Corona und Grippe schützen?

Beide Krankheiten verbreiten sich über die gleichen Ansteckungswege, daher sind auch die Schutzmaßnahmen gleich:

- Abstand halten (mindestens 1,5 Meter)
- Mund-Nasen-Schutz tragen
- Kontakte reduzieren
- Häufiges und gründliches Händewaschen
- Menschenansammlungen und Großveranstaltungen meiden
- Auf Händeschütteln, Umarmungen, etc. verzichten
- Regelmäßig gründlich Lüften
- Treffen mit Freunden eher an der frischen Luft

Auf einen Blick: Unterscheidungsmerkmale Corona und Grippe

Die Virus-Erkrankungen unterscheiden sich in **drei Punkten** voneinander:

1. **Verlauf**: Das erste wichtige Unterscheidungsmerkmal ist das Tempo, in dem sich die Erkrankung ausbildet. Grippesymptome treten meist schlagartig auf, sodass sich die Betroffenen innerhalb weniger Stunden sehr krank fühlen und hohes Fieber entwickeln. Eine Infektion mit dem Corona-Virus verschlimmert sich eher langsam über Tage.
2. **Störung Geschmacks- und Geruchssinn**: Viele Covid-19-Patienten berichten, dass sie nichts mehr schmecken und riechen können. Zwar können ähnliche Symptome auch bei einer starken Erkältung auftreten, dann aber eher schleichend.
3. **Schnupfen**: Bei einer Infektion mit dem Grippe-Virus tritt Schnupfen viel häufiger auf als bei Covid-19.

Aber: Gewissheit kann nur ein Test bieten.

Fazit: Corona ist nicht harmloser als die Grippe

Nach aktuellem Erkenntnisstand ist das Corona-Virus **ähnlich gefährlich**, wie die Grippe. Jedoch fehlt im Vergleich zur Grippe die Grundimmunität der Bevölkerung und ein Impfstoff. Ob Corona tödlicher als die saisonale Influenza ist, können Experten erst sagen, wenn die Pandemie vorbei ist. Zumindest sieht es derzeit danach aus, als würde es die Erkrankung in manchen Fällen **länger dauern, manche haben auch länger Störungen**. Ob diese wieder weggehen, wird die Zeit klären.

10 Punkte für Ihre Sicherheit

Sicher im Krankenhaus – trotz Corona-Pandemie. Helios hat ein 10 Punkte Sicherheitskonzept entworfen, dessen Maßnahmen Erkrankte, Besucher und Mitarbeiter vor Corona schützen. Mit dem Sicherheitskonzept gestalten wir Ihren Aufenthalt im Krankenhaus so sicher wie möglich: Beginnend mit einem individuellen Risikocheck über die strikte Trennung von Infizierten und Nicht-Infizierten bis hin zu kurzen Krankenhausaufenthalten und Videosprechstunden.

Sicherheitskonzept lesen

Dieser Artikel gibt den derzeitigen Wissensstand des zuletzt aktualisierten Datums wieder. Er wird regelmäßig nach den neuesten wissenschaftlichen und medizinischen Kenntnissen aktualisiert.
